# BTLA agonist attenuates Th17-driven inflammation in a mouse model of steroid-resistant asthma

**DOI:** 10.3389/fimmu.2025.1552394

**Published:** 2025-03-28

**Authors:** Christine Quach, Xin Li, Pedram Shafiei-Jahani, Meng Li, Stephen Shen, Doumet Georges Helou, Benjamin P. Hurrell, Pejman Soroosh, Omid Akbari

**Affiliations:** ^1^ Department of Molecular Microbiology and Immunology, Keck School of Medicine, University of Southern California, Los Angeles, CA, United States; ^2^ University of Southern California, Libraries Bioinformatics Service, University of Southern California, Los Angeles, CA, United States; ^3^ Janssen Research and Development, San Diego, CA, United States

**Keywords:** steroid-resistant asthma, neutrophilic asthma, BTLA, HVEM, Th17

## Abstract

**Introduction:**

Steroid-resistant asthma does not respond adequately to corticosteroid treatment. The underlying mechanisms driving corticosteroid resistance remain poorly understood, partly due to the absence of suitable animal models. Identifying the immunomodulatory pathways and mechanisms driving steroid resistance is crucial for developing effective therapies.

**Methods:**

In this study, we screened 58 murine strains exposed to house dust mite and identified that the BXD75 strain exhibited neutrophil-skewed, steroid-resistant asthma and elevated Th17 cells. RNA sequencing of lung CD4^+^ T cells from BXD75 was performed to identify immunomodulatory pathways involved in steroid-resistance. The effects of BTLA agonist treatment were assessed on airway hyperreactivity and lung inflammation.

**Results:**

Transcriptomic analysis revealed increased HVEM expression and decreased BTLA expression, both critical immune regulators associated with stimulatory and inhibitory signaling, respectively. These T cells demonstrated enhanced inflammatory signaling through both canonical and non-canonical NF-κB pathways. BTLA agonist treatment *in vivo* reduced airway hyperreactivity and lung inflammation, while *ex vivo* treatment of Th17 cells induced inhibitory signaling via SHP-1, suppressed NF-κB signaling, reduced cell numbers, and lowered IL-17 levels.

**Discussion:**

Our findings establish BXD75 mice as a model for steroid-resistant asthma and demonstrate that BTLA agonism attenuates airway hyperreactivity and lung inflammation, highlighting it as a potential therapeutic strategy.

## Introduction

Asthma, a chronic respiratory disease that affects approximately 262 million people worldwide, is characterized by airway inflammation, bronchial hyperresponsiveness and lung tissue remodeling (1). Asthma is a heterogeneous disease that presents with different phenotypes and endotypes, each with distinct etiologies. In allergic asthma, the most common form of asthma, exposure to common aeroallergens such as pollen, house dust mite (HDM) and pet dander, precipitates the onset of symptoms (2). Most commonly, allergic asthma is treated by daily preventative inhaled corticosteroid (ICS) therapies and *ad-hoc* bronchodilator therapy which, for many patients, is effective in controlling the disease (3). However, 5-10% of patients have severe disease that does not respond to these established treatments (4). This subset of patients is associated with having a greater burden of disease, and a higher mortality rate (2). Hence, there is an urgent need for alternative strategies to combat steroid-resistant asthma.

Asthma is classified generally as either type-2 (Th2)-high or Th2-low asthma (2, 5). Th2-high asthma is predominantly characterized by eosinophilic airway inflammation, driven by the Th2-associated cytokines interleukin (IL)-4, IL-5 and IL-13 (5). Patients with Th2-high asthma respond well to standard therapies including ICS. However, the pathogenesis of Th2-low asthma is not as well understood, and patients with Th2-low asthma are frequently found to be unresponsive to conventional ICS therapies (2, 5, 6). It has been reported that such patients exhibit neutrophilic, rather than eosinophilic, airway inflammation and that this inflammation is predominantly driven by the Th17-derived cytokines IL-17 and IL-22 (2, 5–7). IL-17 in particular, is both a potent stimulatory signal for the local production of neutrophil chemoattractants and a known neutrophil activator (6, 8), which has been shown to be elevated in bronchial biopsies taken from patients with severe asthma compared to healthy controls (9).

T cell subsets, particularly CD4^+^ helper T cells such as Th17 cells, play a central role in mediating the inflammatory response in asthma (10, 11). T cells require two signals to fully activate: one from the T cell receptor (TCR) recognizing its cognate antigen and one from a co-signaling molecule that can positively or negatively modulate TCR signaling (11, 12). Recently, the co-receptor, HVEM (herpesvirus entry mediator), also known as tumor necrosis factor receptor superfamily 14 (TNFRSF14), has emerged as a potential novel biomarker for asthma, thanks to its reported association with disease severity in asthma patients (13). HVEM is a cell surface receptor that can activate NF-κB signaling and while predominantly expressed on T cells; dendritic cells (DCs), monocytes, B cells, neutrophils, natural killer (NK) cells, and endothelial cells are also known to express the receptor (14, 15). Multiple ligands have been identified that bind HVEM, one of which, LIGHT (homologous to lymphotoxin, exhibits inducible expression and competes with HSV glycoprotein D for binding to herpesvirus entry mediator, a receptor expressed on T lymphocytes), has been identified as an important co-stimulatory molecule implicated in driving inflammatory diseases such as asthma (15–17). Studies have demonstrated that sputum levels of LIGHT positively correlate with the severity of airflow limitation in patients with asthma (18, 19). LIGHT is predominantly a cell surface molecule expressed by activated T cells, immature DCs, activated NK cells, ILCs, macrophages, and neutrophils (14, 17). BTLA (B and T lymphocyte attenuator) is another important ligand for HVEM. Unlike LIGHT, BTLA binds HVEM to suppress immune responses. BTLA is primarily expressed in lymphocytes, with CD4^+^ T cells expressing more BTLA than CD8^+^ T cells. The immunosuppressive effects of BTLA-HVEM interactions are similar to those induced by programmed cell death 1 (PD1) and cytotoxic T lymphocyte antigen 4 (CTLA-4) (12, 20). It has been suggested in several studies that BTLA’s involvement in suppressing allergic airway inflammation is via its role in the regulation of T cell survival (21–23). HVEM engagement can therefore result in both pro- and anti-inflammatory outcomes depending on the specific ligand (LIGHT or BTLA), the unique binding site on HVEM, expression patterns on different cell types, and the configuration of the receptor interaction (14). The delicate equilibrium of the LIGHT-HVEM-BTLA axis plays a pivotal role in controlling T cell activation and influencing disease progression, making targeted manipulation of HVEM signaling an increasingly attractive therapeutic approach for treating inflammatory disorders.

Despite this progress, there remain several challenges preventing the development of effective therapies for steroid-resistant asthma. Strikingly, there is no well-established *in vivo* animal model of neutrophilic asthma which would allow for more rapid identification of biomarkers and expand our current limited understanding of the mechanisms involved in the pathogenesis of steroid-resistant asthma (5, 24). In the present study, we exposed 58 established mouse strains to HDM to induce the development of AHR phenotypes that reflect those observed in the human population. Our analyses revealed that the BXD75 mouse strain exhibits a physiological response to HDM that closely resembles that of patients with neutrophilic asthma. Characterization of HDM-exposed BXD75 mice demonstrated the presence of low Th2, but notably high Th17-driven inflammation, which is associated with steroid resistance. These mice were phenotypically characterized by significant neutrophil infiltration in the lungs, increased AHR, and low systemic IgE levels. Depletion of CD4^+^ T cells, but not CD8^+^ T cells, alleviated AHR and neutrophilic inflammation in BXD75 mice, suggesting a pivotal role of CD4^+^ T cells in orchestrating the disease. Within the lungs, CD4^+^ T cells from BXD75 mice exhibited markedly reduced levels of BTLA, along with significantly elevated levels of HVEM and LIGHT, compared to C57BL/6 controls. By utilizing a BTLA agonist, we demonstrated that therapeutic induction of BTLA inhibitory signaling attenuates excessive Th17-driven inflammation and reduces AHR. Mechanistically, our results suggest that BTLA engagement promotes the recruitment of SHP-1 and suppression of NF-κB signaling. Overall, our findings identify BXD75 mice as a valuable animal model of steroid-resistant asthma and offer new insights into the modulation of T cell activation via co-inhibitory receptor induction, highlighting its potential as a promising therapeutic approach for steroid-resistant asthma.

## Results

### Lung function and inflammation varies in response to HDM exposure among 58 mouse strains

**Figure 1:**
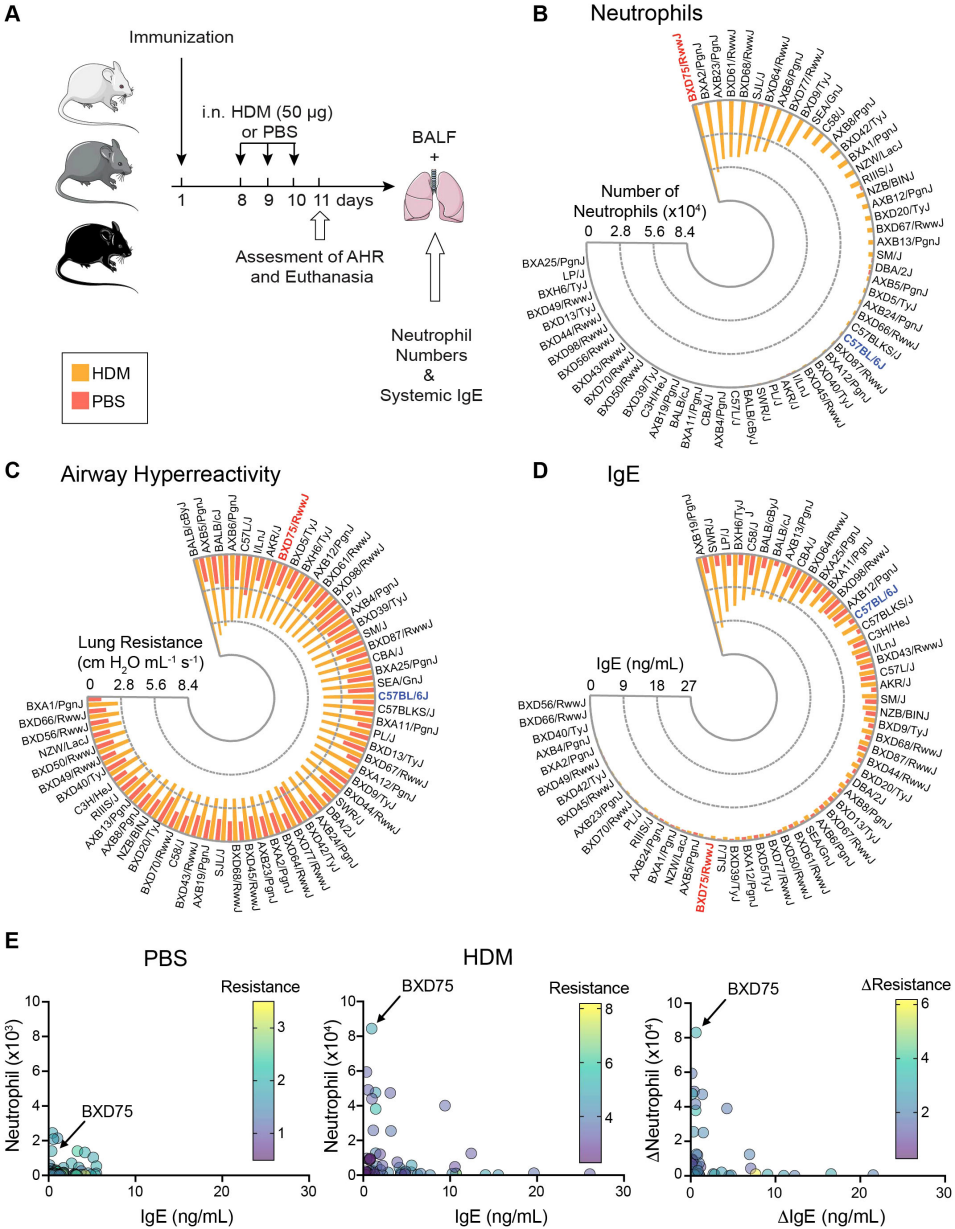
Lung function and inflammation varies in response to HDM exposure among 58 mouse strains. **(A)** 58 mice strains were immunized with 25 μg of HDM in 0.5 mg alum. On Day 8, mice were intranasally challenged with 50 μg of HDM or PBS for 3 consecutive days. On Day 11, lung function (AHR), BALF cellularity, and BALF cytokines were assessed. Radial bar graph of the **(B)** absolute numbers of neutrophils, **(C)** lung resistance at 40 mg/mL methacholine, and **(D)** levels of IgE among 58 mice strains challenged with PBS (orange bars) or HDM (yellow bars). Each bar represents the mean value of 3-4 mice per group per strain. **(E)** Scatterplot of absolute neutrophil numbers, IgE levels and lung resistance at 40 mg/mL methacholine for PBS (left) and HDM (middle). Difference between HDM and PBS exposure (right) in neutrophil number, IgE levels and lung resistance at 40 mg/mL methacholine among 58 mice strains.

New mouse models that can recapitulate the steroid-resistant airway inflammation observed in patients with severe asthma are needed. We previously adapted an established protocol using the Hybrid Mouse Diversity Panel (HMDP) and performed a murine genome wide association study (GWAS) to screen for airway hyperreactivity (AHR) and immune responses to diesel exhaust particles (DEP) (25–27). We observed variable responses between strains in response to DEP exposure. Building on our previous findings, we strategically selected 58 strains exhibiting strong responses to allergens for in-depth phenotypic characterization in response to HDM challenge, a clinically relevant major allergen implicated in asthma. To ensure clinical relevance, we employed an established protocol for HDM challenge which allowed us to systematically evaluate the diverse and immunological and physiological responses across the 58 genetically distinct strains (**Supplementary Table S1**) (28, 29). Mice were immunized with 25 μg of HDM in 0.5 mg alum intraperitoneally (*i.p*.) and one week later were intranasally (*i.n*) challenged with 50 μg of HDM for 3 consecutive days (**Figure 1A**). Twenty-four hours after the final HDM challenge, we evaluated lung function by directly measuring lung resistance in response to increasing methacholine concentrations, followed by bronchoalveolar lavage fluid (BALF) collection for further analysis (**Figure 1A**). Our findings revealed that out of the 58 strains, BXD75 mice remarkably exhibited the highest neutrophil count among all strains tested (**Figure 1B**, **Supplementary Figure S1A**). We then assessed airway hyperreactivity (AHR) by exposing the mice to increasing doses of methacholine, a potent bronchoconstrictor. Although several mouse strains demonstrated high lung resistance, BXD75 mice were the only strain that combined high neutrophilia and AHR levels in the top quartile (**Figure 1C**). Lastly, the production of IgE is promoted by Th2 cytokines such as IL-4 and IL-13 and is a key mediator of Th2 inflammatory response (30). Interestingly, when measuring systemic IgE levels, which are typically lower in neutrophilic asthma, we observed that approximately half of the mouse strains exhibited very low IgE concentrations, including BXD75 mice (**Figure 1D**) (31). Among the 58 strains examined, BXD75 mice were unique in their combination of distinctive characteristics of neutrophilic asthma: high neutrophilia, high AHR, and low levels of IgE (**Figure 1E**). This unique profile strongly suggests a Th2-low asthma phenotype with neutrophilic airway inflammation, making BXD75 mice a potentially valuable model organism for studying this specific asthma endotype. The BXD75 mice strain is derived from crosses between C57BL/6 females and DBA/2J males. We therefore selected the BXD75 strain for further characterization, comparing it with the parental C57BL/6 strain as control mice, which are known to develop a Th2-high, eosinophil-driven asthma phenotype (32). Such analysis will allow us to elucidate the distinct mechanisms underlying neutrophilic inflammation and potentially identify novel therapeutic targets for future treatments.

### HDM induces neutrophil-skewed mixed granulocytic airway inflammation in BXD75 mice

**Figure 2:**
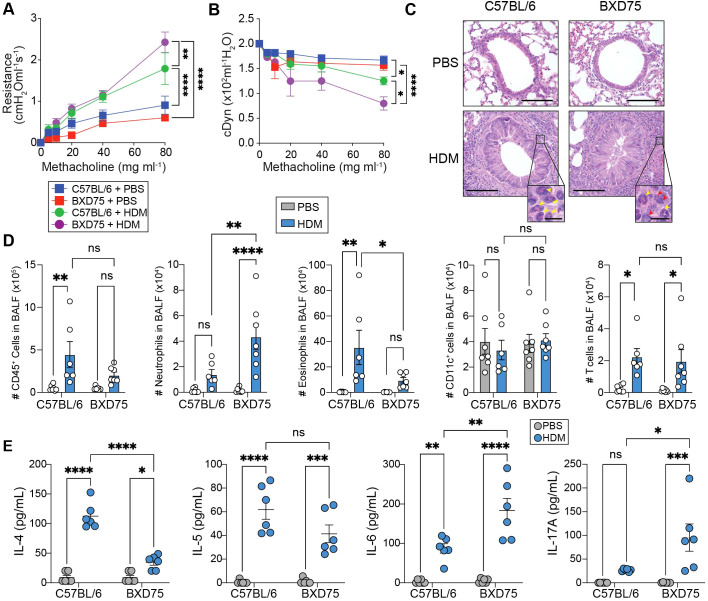
HDM induces neutrophil-skewed mixed granulocytic airway inflammation in BXD75 mice. **(A)** Lung resistance and **(B)** dynamic compliance of C57BL/6 and BXD75 mice challenged with HDM over 3 consecutive days. n = 5-6 mice per group. **(C)** Representative H&E staining of mice lungs. Scale bar, 100 μm. Inset of peribronchial inflammation. Red arrowhead, neutrophils, Yellow arrowhead, eosinophils. Scale bar, 10 μm. **(D)** Total number of CD45^+^ cells, neutrophils (CD45^+^, Ly6G^+^, CD11b^+^), eosinophils (CD45^+^, CD11c^-^, Siglec-F^+^), CD11c^+^ cells (CD45^+^, Ly6G^-^, CD11c^+^), and T cells (CD45^+^, CD3^+^) in the BALF. **(E)** Levels of IL-4, IL-5, IL-6 and IL-17A in the BALF. Data in A and B are from one experiment that is representative of three independent experiments. Data in D and E are pooled from two experiments. Each experiment was performed at least three times. For all quantifications, data are presented as mean ± SEM and analyzed with a two-way ANOVA with Tukey’s multiple comparison test. n.s., not significant; *, *p* < 0.05; **, *p* < 0.01; ***, *p* < 0.001; ***, *p* < 0.0001.

Given our findings that neutrophil populations were significantly elevated in the BALF of BXD75 mice, we wanted to further characterize the BXD75 mice compared with C57BL/6 control by exposing the mice to HDM as previously shown in **Figure 1A**. We found that HDM exposure significantly increased lung resistance compared with PBS controls, regardless of strain background, which was associated with decreased dynamic compliance (**Figures 2A, B**). Consistent with our previous findings, HDM-challenged BXD75 mice exhibited significantly increased lung resistance compared to HDM-challenged control mice (**Figure 2A**). This phenotype was associated with worsened dynamic compliance as a measure of lung elasticity (**Figure 2B**). HDM exposure significantly increased peribronchial inflammation (**Figure 2C**, **Supplementary Figure S1B**). In BXD75 mice, the influx of immune cells surrounding the bronchi were predominantly composed of neutrophils and eosinophils, while in C57BL/6 mice, the peribronchial infiltrate was primarily composed of eosinophils (**Figure 2C**). To assess mucus production, we performed Periodic-acid Schiff (PAS) staining and observed that HDM exposure induced goblet cell hyperplasia as indicated by increased PAS-positive staining compared to PBS controls (**Supplementary Figure S1C**). We next characterized the number and subset of immune cells in the BALF of BXD75 and control mice (**Supplementary Figure S1A**). In confirmation with our previous findings, we observed significantly increased numbers and frequency of neutrophils in the BALF of BXD75 mice compared with C57BL/6 control mice challenged with HDM (**Figure 2D**, **Supplementary Figure S1D**). Although we also detected elevated eosinophils, we found that BXD75 mice harbored significantly fewer BALF eosinophils compared with control mice exposed to HDM (**Figure 2D**, **Supplementary Figure S1D**). Analysis of CD11c^+^ cells, which represent macrophages and dendritic cells, revealed no significant differences between C57BL/6 and BXD75 mice, nor between HDM-challenged and PBS-challenged groups (**Figure 2D**). While HDM challenge significantly increased CD3^+^ T cell numbers compared with PBS control, there was no difference between C57BL/6 and BXD75 mice (**Figure 2D**). Furthermore, HDM challenge increased IL-4, IL-5 and IL-6 in the BALF supernatants compared with PBS challenge regardless of genotype, confirming the Th2 nature of the response generally induced by HDM (**Figure 2E**). Notably, the amount of IL-4 found in the BALF of BXD75 was decreased compared to control mice (**Figure 2E**). Interestingly, however, BXD75 mice had significantly increased IL-17A and IL-6 in the BALF compared to control mice, two cytokines central to Th17-driven inflammation (**Figure 2E**). There were no significant changes in the levels of IFN-γ, a cytokine important in driving Th1 responses, following HDM challenge in either strain (**Supplementary Figure S1E**). Overall, beyond confirming our previous observations suggesting the induction of higher lung resistance and neutrophilia in response to HDM in BXD75 mice, our findings further suggest a Th17-mediated inflammatory response in BXD75 mice compared to C57BL/6 mice.

### Neutrophil-skewed mixed granulocytic airway inflammation in BXD75 mice is resistant to steroid treatment

**Figure 3:**
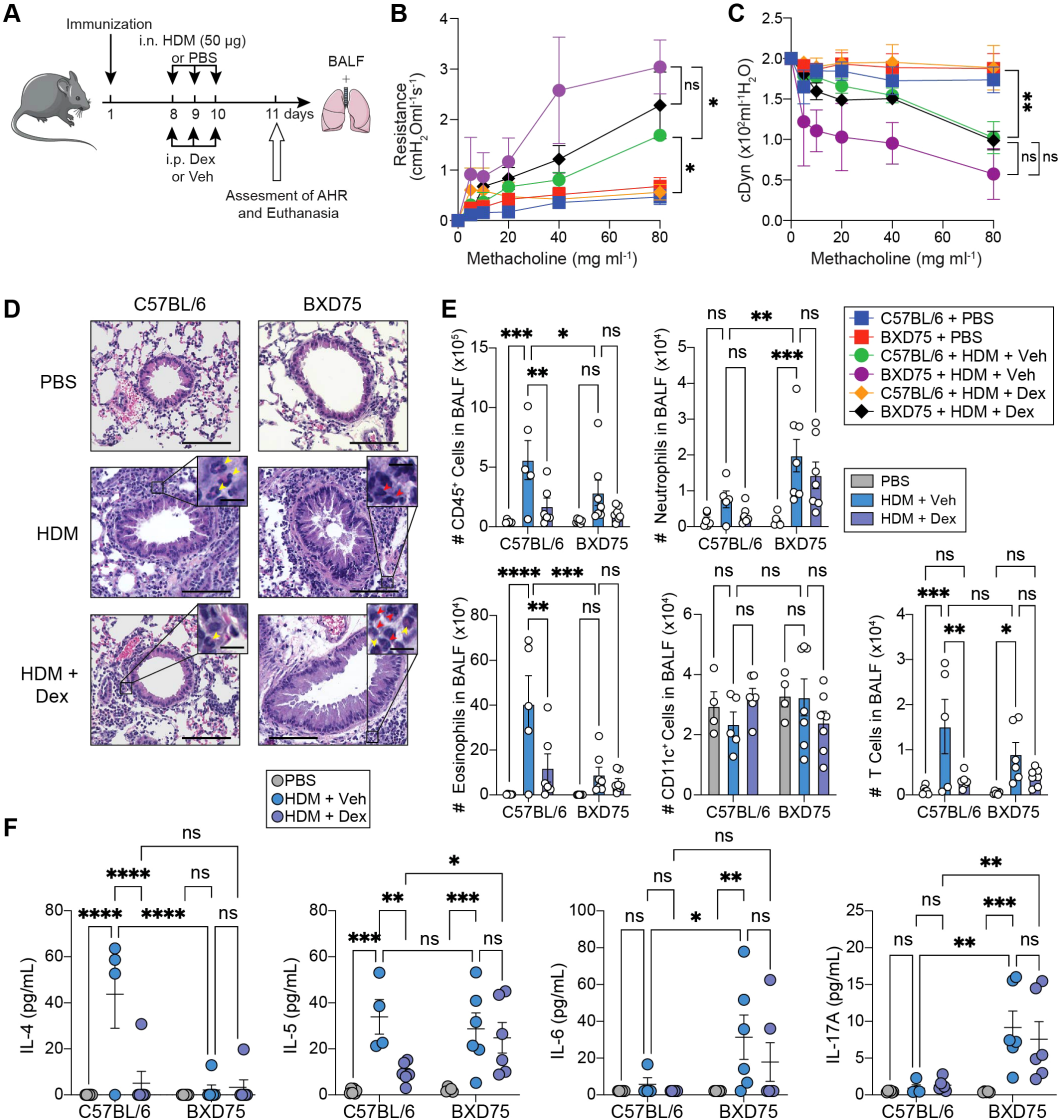
Neutrophil-skewed mixed granulocytic airway inflammation in BXD75 mice is resistant to steroid treatment. **(A)** C57BL/6 and BXD75 were immunized with 25 μg of HDM in alum. On Day 8, mice were intranasally challenged with 50 μg of HDM or PBS for 3 consecutive days and administered dexamethasone (Dex, 1 mg kg^-1^ mouse^-1^) or vehicle (Veh) intraperitoneally. On Day 11, lung function (AHR), BALF cellularity, BALF cytokines histology were analyzed. **(B)** Lung resistance and **(C)** dynamic compliance of C57BL/6 and BXD75 mice challenged with HDM over 3 consecutive days. n = 3-4 mice per group. **(D)** Representative H&E staining of mice lungs. Scale bar, 100 μm. Inset of peribronchial inflammation. Red arrowhead, neutrophils, Yellow arrowhead, eosinophils. Scale bar, 10 μm. **(E)** Total number of CD45^+^ cells, neutrophils (CD45^+^, Ly6G^+^, CD11b^+^), eosinophils (CD45^+^, CD11c^-^, Siglec F^+^), CD11c^+^ cells (CD45^+^, Ly6G^-^, CD11c^+^), and T cells (CD45^+^, CD3^+^) in the BALF. **(F)** Levels of IL-4, IL-5, IL-6 and IL-17A in the BALF. Data in **(B, C)** are from one experiment that is representative of three independent experiments. Data in E and F are pooled from two experiments. Each experiment was performed at least three times. For all quantifications, data are presented as mean ± SEM and analyzed with a two-way ANOVA with Tukey’s multiple comparison test. n.s., not significant; *, *p* < 0.05; **, *p* < 0.01; ***, *p* < 0.001; ***, *p* < 0.0001.

High neutrophil numbers in the sputum of severe asthmatics are generally associated with poor response to steroid treatment (33). We therefore assessed whether C57BL/6 and BXD75 mice were steroid-resistant by testing their response to the glucocorticoid dexamethasone (34). Mice were immunized with HDM and seven days later challenged with HDM and treated with dexamethasone (Dex) or vehicle (Veh) over 3 consecutive days and on day 11 we assessed lung function and inflammation (**Figure 3A**). As expected, we found that HDM-challenged C57BL/6 control mice treated with dexamethasone had significantly decreased AHR as evidenced by reduced lung resistance and increased dynamic compliance compared with mice treated with vehicle (**Figures 3B, C**). Lung resistance and dynamic compliance in Dex-treated control mice, even at its highest nebulized dose, were notably comparable to the values of PBS-treated control mice (**Figures 3B, C**). Remarkably however, dexamethasone treatment in HDM-challenged BXD75 mice had no significant effects on AHR compared to vehicle-treated BXD75 mice, together suggesting a resistance to steroids (**Figures 3B,C**). As expected, dexamethasone reduced peribronchial immune cell infiltration in HDM-challenged C57BL/6 mice, but consistent with our previous findings had no significant effects on the airways of HDM-challenged BXD75 mice compared to vehicle-treated controls (**Figure 3D**, **Supplementary Figure S2A**). PAS staining demonstrated that dexamethasone treatment reduced goblet cell hyperplasia in C57BL/6 mice but had no effect in BXD75 mice (**Supplementary Figure S2B**). Similarly, dexamethasone treatment significantly reduced the number and frequency of eosinophils, as well as the number of T cells in the BALF of HDM-challenged C57BL/6 mice (**Figure 3E**, **Supplementary Figure S2C**). However, it did not significantly affect neutrophil number and frequency or T cell numbers in the BALF of HDM-challenged BXD75 mice (**Figure 3E**, **Supplementary Figure S2C**). The treatment notably had no significant effects on the number CD11c^+^ cells in mice of either background challenged with HDM (**Figure 3E**). Finally, dexamethasone treatment significantly decreased Th2-associated cytokines IL-4 and IL-5 in the BALF of HDM-challenged control mice but had remarkably no effects on Th17-associated cytokines IL-17A and IL-6 in BXD75 mice (**Figure 3F**). The levels of CXCL1 (KC), a chemokine important for neutrophil recruitment, were significantly elevated in the BALF of BXD75 mice exposed to HDM compared with C57BL/6 mice (**Supplementary Figure S2D**). HDM challenge had no significant effects on the levels of IFN-γ in both mouse strains (**Supplementary Figure S2D**). Similarly, dexamethasone treatment had no effects on the levels of CXCL1 or IFN-γ. Together, our findings therefore suggest that although dexamethasone reduced airway inflammation characteristics in C57BL/6 mice, it had no effects on BXD75 mice during HDM-induced inflammation. Dexamethasone did not affect AHR, neutrophilia, Th17-driven cytokines or goblet cell hyperplasia, in BXD75 mice following HDM exposure, together suggesting a resistance to steroids in these mice.

### HDM exposure induces Th17 gene expression in lung CD4^+^ T cells from BXD75 mice

T cells, particularly CD4^+^ T helper cells, play a crucial role in the development and progression of asthma by contributing to airway inflammation, AHR, and tissue remodeling. Therefore, to identify the T cell subset predominantly involved in asthma pathogenesis, we selectively depleted CD4^+^ or CD8^+^ T cells in C57BL/6 mice challenged with HDM. (**Supplementary Figure S3A**). The neutralizing antibodies against CD4 and CD8 specifically and effectively depleted their respective targets (**Supplementary Figure S3B, C**). As anticipated, CD4^+^ T cell depletion, but not CD8^+^ T cell depletion, significantly reduced lung resistance and improved dynamic compliance (**Supplementary Figures S3D, E**). Consistently, only CD4^+^ T cell depletion reduced the numbers of neutrophils and eosinophils in the BALF of HDM challenged mice, while neither CD4 nor CD8 depletion affected CD11c^+^ cell numbers (**Supplementary Figure S3F**). These findings demonstrate that CD4^+^ T cells are essential mediators of airway hyperreactivity and lung inflammation in BXD75 strain.

**Figure 4:**
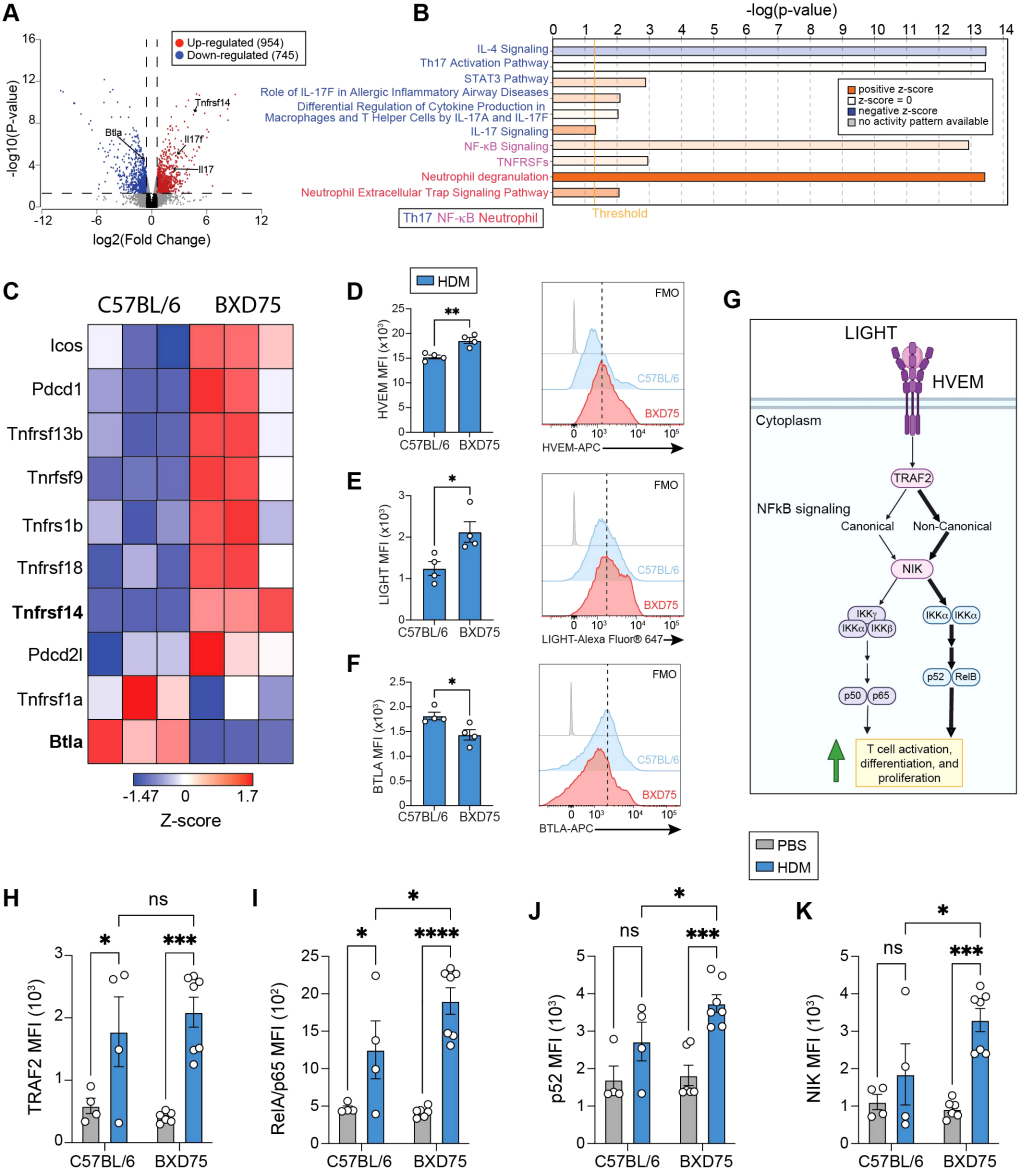
HDM-driven airway inflammation in BXD75 mice are enriched for genes related to Th17 mediated inflammation. **(A)** Volcano plot of differentially expressed genes (DEGs) in C57BL/6 vs BXD75 mice challenged with HDM only. **(B)** Gene set enrichment analysis by Ingenuity Pathway Analysis (IPA) depicting enriched pathways in HDM-challenged BXD75 mice. **(C)** Heatmap representation of co-receptors in CD4^+^ T cells isolated from BXD75 mice vs C57BL/6 mice challenged with HDM. n = 3 mice per genotype. Mean fluorescence intensity (MFI) and representative flow cytometry histograms of **(D)** HVEM, **(E)** LIGHT, and **(F)** BTLA in CD4^+^ T cells from C57BL/6 and BXD75 mice challenged with HDM. **(G)** Schematic of downstream HVEM-LIGHT stimulatory signaling in T cells. MFI of **(H)** TRAF2, **(I)** p65, **(J)** p52, and **(K)** NIK in CD4^+^ T cells from HDM or PBS challenged C57BL/6 and BXD75 mice. Data in **(D–F)** are from one experiment that is representative of three independent experiments. Data in **(H–K)** are pooled from two experiments. Each experiment was performed at least three times. For all quantifications, data are presented as mean ± SEM and analyzed with a two-way ANOVA with Tukey’s multiple comparison test. n.s., not significant; *, *p* < 0.05; **, *p* < 0.01; ***, *p* < 0.001; ***, *p* < 0.0001.

To gain mechanistic insights, we therefore analyzed the transcriptome of CD45^+^CD3^+^CD4^+^ T cells isolated from the lungs of C57BL/6 and BXD75 mice challenged with HDM as previously described (**Figure 1A**). Comparative analysis of gene expression profiles identified 1699 differentially expressed genes (DEGs) between BXD75 and C57BL/6 mice challenged with HDM (**Figure 4A**). Amongst these, 954 transcripts were upregulated in BXD75 mice, which notably included Th17 key cytokines *il17a* and *il17f* (**Figure 4A**). Subsequent pathway analysis of the DEGs was performed using Ingenuity Pathway Analysis (IPA). This analysis revealed a significant upregulation of genes associated with Th17 differentiation and activation including genes related to STAT3 and IL-17 signaling and downregulation of genes related to IL-4 signaling in HDM-challenged BXD75 mice compared with HDM-challenged C57BL/6 control mice (**Figure 4B**). Core genes that drive Th17 differentiation, such as *Rorc, Rora, il17a, il17f*, and *Ccr6* were upregulated in T cells isolated from HDM-challenged BXD75 mice compared with HDM-challenged C57BL/6 mice (**Supplementary Figure S4**). Similarly, transcripts that negatively regulate Th17 differentiation and activity, including *Il10, Il4, Stat4, Ccl1*, and *Ifng*, are significantly downregulated in T cells isolated from BXD75 mice compared to controls (**Supplementary Figure S4**). Additionally, genes involved in neutrophil activation pathways were upregulated in CD4^+^ T cells isolated from the lungs of the BXD75 strain compared to control mice (**Figure 4B**). These findings are consistent with the observed neutrophilic-skewed inflammation and suggest a shift towards a Th17-mediated immune response in the BXD75 mice following HDM challenge. Interestingly, genes related to the TNF receptor superfamily (TNFRSF) were upregulated in T cells isolated from BXD75 mice, which was supported in parallel by the upregulation of downstream NF-κB signaling pathway (**Figure 4B**). Therefore, we screened the expression of co-receptors such as TNFRSFs, which are known central modulators of CD4^+^ T cell function. We notably found an upregulation in genes of the TNFRSF including *Tnfrsf14, Tnfrsf13b, Tnfrsf9, Tnfrsf1b, Tnfrsf18 and Tnfrsf1a* in CD4^+^ T cells from BXD75 mice (**Figure 4C**). Among all TNFRSF members however, *Tnfrsf14*, encoding for HVEM, was the most significantly upregulated in CD4^+^ T cells isolated from BXD75 mice (**Figures 4A-C**). HVEM is an important co-receptor in immune regulation and cell signaling, highlighting its potential implication in the Th17-driven inflammation observed in BXD75 mice. Since HVEM can bind to both BTLA and LIGHT, we closely monitored the expression of these two ligands both at the transcriptomic and protein level on CD4^+^ T cells. Although *Tnfsf14* transcripts, encoding for LIGHT, did not show differential expression at the transcriptomic level between BXD75 and C57BL/6 mice in CD4^+^ T cells, we remarkably found that *Btla*, encoding for BTLA, was significantly decreased in BXD75 compared to control CD4^+^ T cells (**Figure 4C**). To confirm expression at the protein level, we next measured HVEM, LIGHT and BTLA expression by flow cytometry in CD4^+^ T cells isolated from C57BL/6 and BXD75 challenged with HDM. In confirmation of our transcriptomic analysis, we observed significantly increased expression of HVEM in CD4^+^ T cells from BXD75 mice (**Figure 4D**). Although LIGHT was increased in CD4^+^ T cells from BXD75 mice compared to controls, we further found a decreased expression in BTLA in CD4^+^ T cells from BXD75 mice, confirming our transcriptomic analysis (**Figures 4E, F**). Collectively, these findings suggest a potential shift towards a Th17-driven inflammatory response that may be influenced by the LIGHT-HVEM-BTLA signaling axis in the BXD75 mice.

### CD4^+^ T cells from BXD75 mice display enhanced inflammatory signaling through NF-kB pathways

Mechanistically, HVEM-LIGHT signaling primarily activates the canonical NF-κB signaling pathway which is important for T cell differentiation and function (35). However, reports show that it can also activate the non-canonical NF-κB pathway in the context of chronic inflammation (14, 36, 37) (**Figure 4G**). We, therefore, measured the expression of both canonical and non-canonical NF-κB related proteins In CD4^+^ T cells isolated from the lungs of C57BL/6 and BXD75 mice challenged with HDM or PBS. We first observed that HDM challenge increased the expression of TNF receptor-associated factor 2 (TRAF2), which is involved in both canonical and non-canonical NF-κB pathways, regardless of the strain of mouse treated with PBS (**Figure 4H**). Regarding the canonical NF-κB signaling, we found that although HDM challenge upregulated p65 in CD4^+^ T cells of both strains, it was higher in CD4^+^ T cells from HDM-challenged BXD75 compared to controls (**Figure 4I**). Similarly, CD4^+^ T cells isolated from HDM-challenged BXD75 mice exhibited significantly increased expression of NF-κB-inducing kinase (NIK) and p52, key components of the non-canonical NF-κB signaling pathway, compared to C57BL/6 controls (**Figures 4J, K**). Together, these findings therefore suggest that the enhanced HVEM-LIGHT signaling in T cells isolated from BXD75 may significantly induce NF-κB pathways.

### BTLA agonist ameliorates HDM-induced lung inflammation

**Figure 5:**
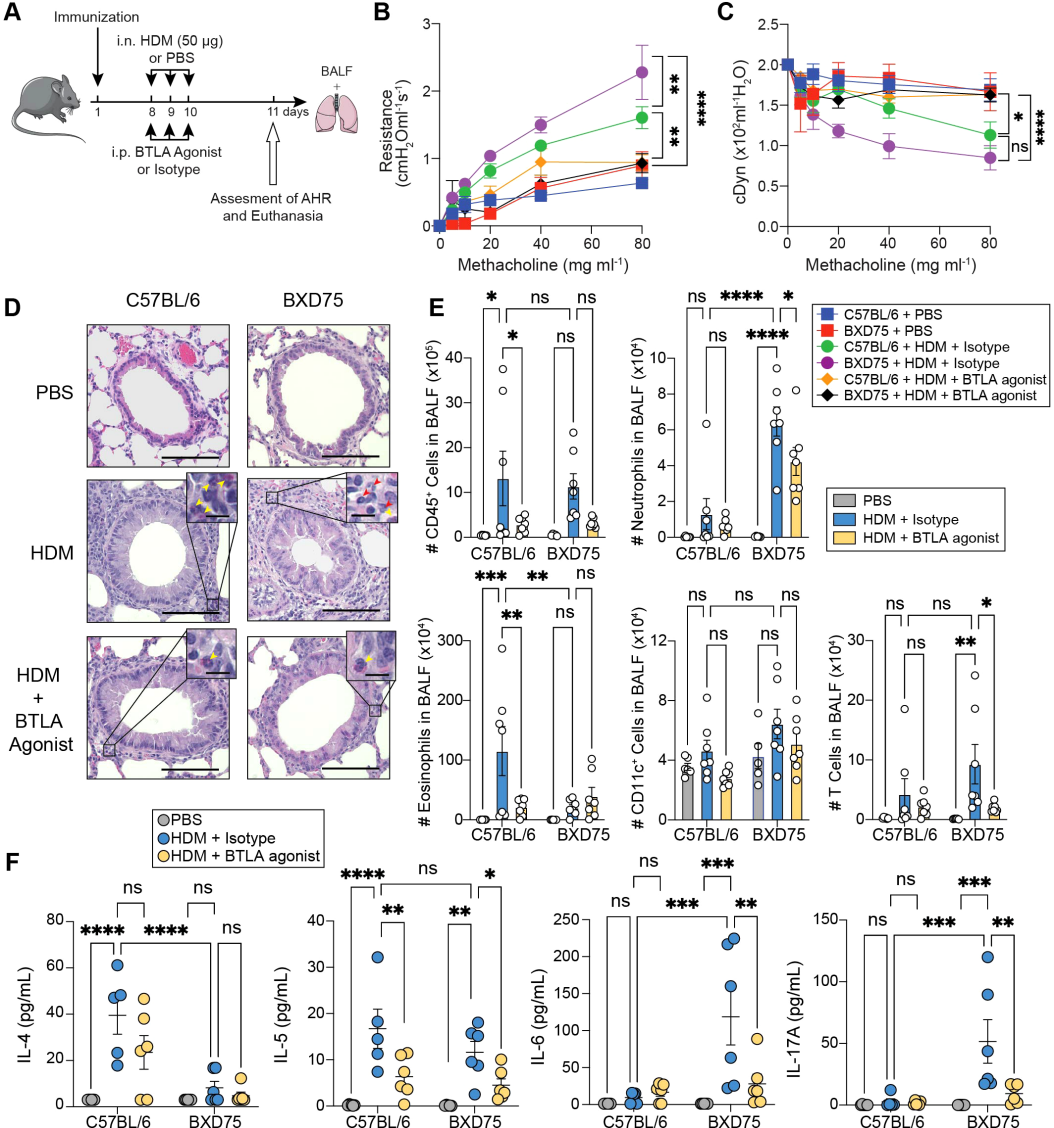
BTLA agonist ameliorates HDM induced lung inflammation. **(A)** C57BL/6 and BXD75 were immunized with 25 μg of HDM in alum. On Day 8, mice were intranasally challenged with 50 μg of HDM or PBS for 3 consecutive days and administered BTLA agonist (Clone 6A6, 100 μg mouse per day) or Isotype intraperitoneally. On Day 11, lung function (AHR), BALF cellularity, BALF cytokines histology were analyzed. **(B)** Lung resistance and **(C)** dynamic compliance of C57BL/6 and BXD75 mice challenged with HDM and administered BTLA agonist or Isotype over 3 consecutive days. n = 4-6 mice per group. **(D)** Representative H&E staining of mice lungs. Scale bar, 100 μm. Inset of peribronchial inflammation. Red arrowhead, neutrophils, Yellow arrowhead, eosinophils. Scale bar, 10 μm. **(E)** Total number of CD45^+^ cells, neutrophils (CD45^+^, Ly6G^+^, CD11b^+^), eosinophils (CD45^+^, CD11c^-^, Siglec-F^+^), CD11c^+^ cells (CD45^+^, Ly6G^-^, CD11c^+^), and T cells (CD45^+^, CD3^+^) in the BALF. **(F)** Levels of IL-4, IL-5, IL-6 and IL-17A in the BALF. Data in **(B, C)** are from one experiment that is representative of three independent experiments. Data in **(E, F)** are pooled from two experiments. Each experiment was performed at least three times. For all quantifications, data are presented as mean ± SEM and analyzed with a two-way ANOVA with Tukey’s multiple comparison test. n.s., not significant; *, *p* < 0.05; **, *p* < 0.01; ***, *p* < 0.001; ***, *p* < 0.0001.

BTLA functions as a negative regulator of T cell responses by inhibiting T cell receptor (TCR)-mediated activation and subsequent proliferation (38). It was previously reported that IL-5 production and eosinophilic airway inflammation were increased in the airways of BTLA deficient mice (22). Since our results indicate a reduction of BTLA expression in T cells isolated from BXD75 mice, we hypothesized that inducing the inhibitory effects of BTLA with an agonist could attenuate airway inflammation in these mice during HDM challenge. We therefore immunized C57BL/6 and BXD75 mice with HDM and seven days later challenged with HDM, concurrently with 100 μg BTLA agonist (clone 6A6) *i.p.*, previously reported to attenuate Graft-versus-host-disease (GVHD), or isotype control (**Figure 5A**) (39). Twenty-four hours after the last HDM challenge and BTLA agonist or isotype treatment, we assessed lung function and inflammation. As expected, mice challenged with HDM generally exhibited higher AHR compared to PBS-controls (**Figure 5B**). However remarkably, BTLA agonist treatment significantly decreased AHR in both BXD75 and control mice challenged with HDM, as evidenced by decreased lung resistance and increased dynamic compliance compared with mice treated with isotype control (**Figures 5B, C**).

BTLA agonist treatment reduced the influx of immune cells into the lung tissue of mice challenged with HDM (**Figure 5D**, **Supplementary Figure S5A**). Treatment with BTLA reduced peribronchial inflammation and goblet cell hyperplasia in both BXD75 and C57BL/6 mice (**Supplementary Figures S5A, B**). Flow cytometry analysis of immune cells in the BALF revealed a significant decrease in the total numbers of immune cells in the BALF following BTLA agonist treatment in both C57BL/6 and BXD75 mice (**Figure 5E**). We did not detect differences in neutrophils, CD11c^+^ cells or T cells, but notably found a significant drop in the number and frequency of eosinophils in C57BL/6 mice (**Figure 5E**, **Supplementary Figure S5C**). In BXD75, the number and frequency of eosinophils, as well as CD11c^+^ cell number were not affected by BTLA treatment, but we remarkably found a decrease in both neutrophils and T cells following BTLA agonist treatment (**Figure 5E**, **Supplementary Figure S5C**). In alignment with these results, there was a significant decrease in the levels of IL-5 in the BALF of HDM-challenged C57BL/6 mice but not IL-4, whereas BTLA agonist treatment significantly decreased Th17-associated cytokines IL-17A and IL-6 as well as CXCL1 levels in BXD75 mice (**Figure 5F**, **Supplementary Figure S5D**). BTLA agonist treatment did not affect IFN-γ levels (**Supplementary Figure S5D**). These findings, therefore, highlight BTLA induction as a potential target for not only treating Th2 high eosinophilic airway inflammation but also steroid-resistant airway inflammation associated with Th17 type inflammation.

### BTLA agonist treatment attenuates Th17 activation *ex vivo*

**Figure 6:**
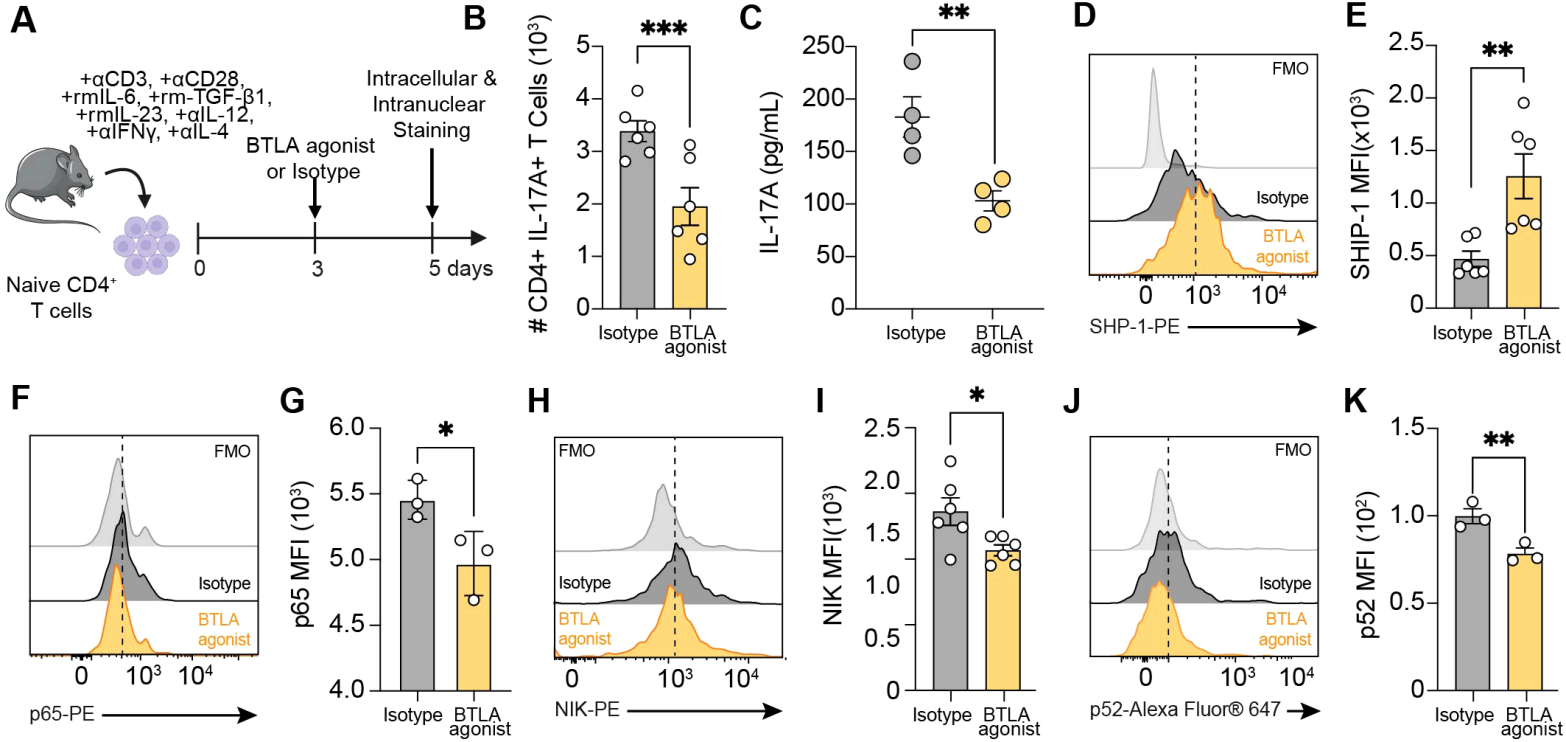
BTLA agonist treatment attenuates Th17 activation *in vitro*. **(A)** Naïve CD3^+^CD4^+^CD44^low^ T cells were isolated from C57BL/6 mice and cultured with bound anti-CD3, soluble CD28, rmIL-6, rmTGF- β1, rmIL-23, anti-IL-12, anti-IL-4 and anti-IFNγ for 5 days. On day 3, cells were treated with 20 μg/mL of BTLA agonist (Clone 6A6) or Isotype. **(B)** The number of CD4^+^IL^-^17A^+^ T cells derived from C57BL/6 mice. **(C)** Levels of IL-17A in the cell culture supernatant. **(D)** Representative flow cytometry histogram and **(E)** quantification of SHP-1 expression MFI on Day 5. **(F)** Representative flow cytometry histogram and **(G)** quantification of p65 expression MFI on Day 5. **(H)** Representative flow cytometry histogram and **(I)** quantification of NIK expression MFI on Day 5. **(J)** Representative flow cytometry histogram and **(K)** quantification of p52 expression MFI on Day 5. Histograms are from one experiment that is representative of three independent experiments. Data in **(B, C, E, I)** are pooled from two experiments. Data in **(G, K)** are from one representative experiment. Each experiment was performed at least three times. For all quantifications, data are presented as mean ± SEM and analyzed with a two-tailed Student’s *t* test. n.s., not significant; *, *p* < 0.05; **, *p* < 0.01; ***, *p* < 0.001; ***, *p* < 0.0001.

Based on our observation that BTLA agonist treatment *in vivo* reduces neutrophil recruitment to the lungs and lowers IL-17 levels in the BALF, we aimed to investigate the mechanisms by which BTLA activation contributes to attenuating airway inflammation. We isolated naïve CD3^+^CD4^+^CD44^low^ T cells from naïve C57BL/6 mice by FACS and cultured them in Th17 activation media containing anti-CD3, CD28, rmIL-6, rmIL-23, rmTGF-β1, anti-IL12, anti-IL-4 and anti-IFNγ for 5 days (**Figure 6A**) (40, 41). On Day 3 of culture, cells were treated with 20 μg/mL of BTLA (Clone 6A6) or isotype control and on day 5 cells were collected for intracellular and intranuclear staining for Th17 differentiation and BTLA signaling (**Figure 6A**). Flow cytometric analysis revealed that BTLA agonist treatment significantly reduced the frequency of CD4^+^IL-17^+^ T cells compared to isotype controls (**Figure 6B**). Consistent with this observation, BTLA agonist treatment significantly decreased IL-17A levels in T cell cultures (**Figure 6C**). These results demonstrate that BTLA activation effectively suppresses Th17 differentiation and IL-17A production in Th17 cells, suggesting an inhibitory role of BTLA signaling in Th17 cell development.

Mechanistically, BTLA-HVEM binding results in the recruitment of Src homology domain 2 (SH2)-containing protein tyrosine phosphatases, SHP-1 and SHP-2 (**Supplementary Figure S6**) (38). Previous reports suggest that BTLA preferentially recruits SHP-1 over SHP-2 and that SHP-1 recruitment inhibits both CD28 and CD3 phosphorylation whereas SHP-2 inhibits CD28 phosphorylation (42). This results in the inhibition of TCR signaling by modulating key signaling molecules, including NF-kB, which subsequently suppresses T cell activation and proliferation (43). For example, BTLA overexpression has been reported to induce an inhibitory effect on the phosphorylation of IκB and p65 (44). SHP-1 also dephosphorylates P13K leading to reduced Akt phosphorylation which results in suppression of proliferation and cell survival (45). In support of these findings, we found that BTLA agonist increased the expression of SHP-1 (**Figures 6D, E**). We next measured the capacity of the BTLA agonist to modulate NF-κB signaling. Consistent with our findings that T cells from BXD75 mice exhibited higher p65 and p52 expression (**Figures 4H-K**), we found that BTLA agonist treatment decreased the canonical NF-κB protein, p65, and non-canonical NF-κB proteins, NIK and p52, compared to isotype controls (**Figures 6F-K**). Taken together, our observations therefore suggest that BTLA agonist treatment significantly decreased Th17-mediated inflammation, likely through both the canonical and non-canonical NF-κB pathways (**Supplementary Figure S6**). Our data further identifies an inhibitory role of BTLA signaling in Th17 development.

## Discussion

In this study, we established a murine model of steroid-resistant asthma to identify new therapeutic targets for effective treatments. The lack of effective treatments for steroid-resistant asthma can be attributed to various factors including limited knowledge, complex immunophenotypes due to asthma heterogeneity and lack of relevant animal models. Herein, we exposed 58 strains of mice to HDM and identified the BXD75 strain to exhibit a Th2-low phenotype consistent with what is observed in human neutrophilic asthma, a characteristic previously undescribed in this strain (18, 31, 33, 46). We confirmed that BXD75 mice had the highest BALF neutrophil numbers, increased AHR, low levels of IgE and steroid resistance. Interestingly, approximately half the mice strains exhibited low levels of IgE, an important player in Th2-driven inflammatory responses. IgE levels typically peak at around 12-14 days post allergen exposure (47). Our acute sensitization model concludes on day 10, likely capturing only the early phase of IgE production. However, it is important to clarify that IgE levels across all strains, along with two other criteria, were measured to exclude high responders when selecting the neutrophilic asthma model. Additionally, genetic variations among the mouse strains could significantly influence IgE production and Th2-mediated inflammatory response. The BXD75 mice had a Th17-driven inflammatory response which was previously demonstrated to mediate neutrophilic airway inflammation and subsequent steroid-resistance (34, 48, 49). Transcriptomic analysis indicated that T cells isolated from the lungs of BXD75 mice exhibited increased expression of genes encoding several TNF-receptor superfamily proteins, notably HVEM. We demonstrated that inducing HVEM-BTLA inhibitory signaling attenuated airway inflammation, reduced goblet cell hyperplasia, and reduced AHR *in vivo* and attenuated Th17 immune response *ex vivo*, therefore, supporting its therapeutic potential in treating steroid-resistant asthma.

Neutrophilic asthma is characterized by an increase in neutrophils, often with lower eosinophil counts compared to other asthma phenotypes such as eosinophilic asthma and presence of IL-17 and steroid resistance (50, 51). Although eosinophils are present, they are usually at lower levels and may diminish as neutrophilic inflammation persists. However, studies have shown that patients with mixed granulocytic asthma, characterized by both elevated levels of neutrophils and eosinophils, experience greater loss of lung function and exhibit steroid resistance (18, 52, 53). This adds to the complexity of developing murine models that accurately recapitulate that pathophysiology of human steroid-resistant asthma. Several models have been developed to study steroid-resistant severe asthma. Many of these models incorporate microbial components to drive neutrophil recruitment in contrast to our model that uses aluminum hydroxide, a Th2-skewing adjuvant that enhances eosinophilic airway inflammation. One commonly used model combines allergens such as HDM or ovalbumin (OVA) with Complete Freund’s Adjuvant (CFA), which contains heat-killed mycobacterium tuberculosis. HDM in CFA induces a neutrophilic airway inflammation with minimal eosinophil involvement (54). While eosinophilic inflammation is a dominant feature of OVA-based models, the addition of lipopolysaccharides (LPS) shifts the response towards a mixed granulocytic phenotype, characterized by neutrophil-driven airway inflammation (55). While OVA models are classical models in asthma research due to their ability to induce robust immune responses, they may not fully replicate the chronic inflammation and underlying mechanisms of human asthma. HDM combined with the bacterial-derived adjuvant, cyclic di-GMP (CDG), also induces neutrophil dominant airway inflammation (56). Lastly, the fungal allergen *Alternaria alternata* has been used in combination with CFA to induce a robust neutrophilic-dominant airway inflammation (57). These models demonstrate either Th17, Th1 or Th1/Th17 driven airway inflammation and show a higher frequency of neutrophils compared with eosinophils in the airways. Interestingly, in addition to elevated eosinophils, BXD75 mice had elevated neutrophils after HDM challenge compared with C57BL/6 mice. Therefore, one limitation of our study is that our HDM in alum model is an acute model of asthma which does not fully reflect the chronic nature of human asthma. The elevated eosinophil count may be attributed to the HDM-alum combination. It is possible that HDM alone or in use with other adjuvants may affect relative proportions of eosinophils and neutrophils. Therefore, future directions will explore whether other adjuvants or the allergen alone affect the relative proportions of eosinophils and neutrophils. The significance of the BXD75 mouse model is in the strain’s response to HDM which is known to induce a Th2-high eosinophilic airway inflammation in both humans and mouse models of asthma (58). Despite elevated eosinophil levels, BXD75 mice develop Th2-low neutrophilic-skewed mixed granulocytic airway inflammation driven by a Th17 response compared with C57BL/6 mice with Th2-high eosinophilic airway inflammation. Additionally, while Th17 type inflammatory response are often implicated in steroid resistance in severe asthma, some studies suggests that other pathways may also contribute (56). Therefore, future studies using IL-17 neutralization or genetic knockout approaches would be instrumental in confirming the role of IL-17 in driving steroid-resistant inflammation in BXD75 mice exposed to HDM. Our discovery clearly highlights the heterogeneity of asthma due to the complex interactions between environmental exposure and genetic factors. Based on our findings, future studies are warranted to establish the genetic alterations causing these different immune responses to the same allergen. These include studies aimed at identifying key SNPs in BXD75 mice which may reveal a susceptibility gene to steroid-resistant asthma, mirroring how human genetic and environmental factors shape asthma endotypes.

T cell subsets, particularly T helper cells, play a crucial role in orchestrating the type of immune response in asthma (59). Therefore, targeting these specific cells and modulating their activity could reduce or eliminate inflammation in asthma leading to improved lung function. T cell activation and differentiation into specific subsets are not only dependent on TCR-Major Histocompatibility Complex (MHC) interactions, but also on secondary signals mediated by co-signaling molecules that carefully regulate T cell activation, differentiation, survival and cytokine production (12, 60). Co-signaling molecules can deliver stimulatory or inhibitory signals that play a pivotal role in maintaining immune homeostasis. We previously demonstrated that targeting the co-inhibitory receptor Programmed Cell Death Protein 1 (PD-1) with an agonist could alleviate neutrophilic asthma by decreasing neutrophil recruitment, downregulating T effector cells, decreasing AHR, and modulating cytokine production (54). We also demonstrated that PD-1 agonist could ameliorate AHR and suppress Th2 inflammatory responses through regulating type 2 innate lymphoid cells (ILC2s), which are key players in type 2 responses to allergens in the lungs (61). In support of these findings, it has been recently demonstrated that inhibition of PD-1 enhances T cell proliferation and production of Th2 and Th1 cytokines in response to aeroallergens (62). These studies therefore highlight the potential of targeting co-inhibitory receptors in asthma.

There are limited studies highlighting the important role of HVEM in asthma pathophysiology. Interestingly, HVEM can act as a bidirectional switch to provide both activating and inhibitory signals depending on its binding partner (14). The intricate interplay and balance of interactions within the LIGHT-HVEM-BTLA signaling axis may therefore play a crucial role in the development of severe neutrophilic asthma in BXD75 mice as demonstrated in a different context by others (63, 64). Given that HVEM is involved in modulating T cell activity and that T cells play a crucial role in asthma, it is highly likely that modulating the balance of the LIGHT-HVEM-BTLA signaling axis could affect asthma progression, particularly in steroid-resistant asthma. The transcriptomic data of CD4^+^ T cells from BXD75 mice revealed elevated *Tnfsrf14* transcripts, encoding HVEM, which was confirmed to be elevated at the protein level. Promisingly, *Tnfsrf14* transcripts in the blood have been found to be significantly higher in severe and moderate persistent asthma compared with mild and healthy control, illustrating its potentially important role (13). Our transcriptomic analysis revealed decreased *Btla* transcripts in T cells from BXD75 mice, which was confirmed to be significantly decreased at the protein level in comparison to C57BL/6 control mice. Interestingly however, while no significant differences were observed in the transcript levels of *Tnfsf14* (the gene encoding LIGHT), the LIGHT protein itself was found to be significantly elevated in T cells from BXD75 mice. This discrepancy between mRNA and protein levels suggests potential post-translational regulation or increased protein stability in BXD75 mice. We propose that the reduced BTLA expression on T cells from BXD75 mice disrupts the equilibrium of the LIGHT-HVEM-BTLA axis. In the absence of adequate BTLA to interact with HVEM, this receptor might become more accessible to other binding partners, including LIGHT. Such increased availability could potentially lead to enhanced T cell activation, as previously described in studies (65, 66). Consequently, the combination of reduced inhibitory signaling and potentially increased activating signaling due to high HVEM and LIGHT levels on CD4^+^ T cells of BXD75 mice could create a perfect storm for the development and persistence of steroid-resistant airway inflammation.

LIGHT-HVEM signaling activates both the canonical and non-canonical NF-κB signaling pathway (14, 67). Our data demonstrated significantly increased expression of both canonical and non-canonical associated proteins p65/RelA and p52 respectively in CD4^+^ T cells from HDM-challenged BXD75 mice compared with control mice. This heightened NF-κB signaling suggests a highly inflammatory state in T cells from HDM-challenged BXD75 mice compared to control mice consistent with a dysregulated LIGHT-HVEM-BTLA axis. In support of our findings, previous studies have shown that BTLA induction suppresses NF-κB signaling in T cells (44, 66, 68). For example, one study reported that p65/RelA and c-Rel drove Th17 differentiation and response through activation of Th17-associated transcription factor RORγt and RORγ (69). Additionally, non-canonical NF-κB signaling has been associated with Th17 differentiation and effector function through induction of GM-CSF (70, 71). Furthermore, NIK knockout mice were defective in Th17 differentiation in an experimental autoimmune encephalomyelitis mouse model (72). Previous data and our findings therefore highlight a role of both canonical and non-canonical NF-κB signaling in mediating Th17 differentiation in BXD75 mice.

The BTLA pathway plays an important role in negatively regulating immune cell activation and proliferation to prevent excessive inflammation. Therefore, dysregulated BTLA signaling can be implicated in wide range of human diseases. Studies have shown that BTLA expression is often reduced in patients with severe asthma or autoimmune diseases (73). Overall, the BTLA pathway is critical for maintaining immune homeostasis in many disease contexts. Recent research has highlighted the potential of the HVEM-BTLA pathway as a promising target for cancer immunotherapy, autoimmune diseases and inflammatory disorders (23, 71, 74, 75). A strong correlation between BTLA expression and both asthma severity and IL-17 levels was recently reported (76). Preclinical studies and early-phase clinical trials have demonstrated that BTLA agonists can reduce airway inflammation, improve lung function, and decrease T cell numbers in animal models of asthma, with initial human studies suggesting beneficial effects on controlling inflammation and improving asthma symptoms (21, 39, 77). The BXD75 mice may have dysregulated LIGHT-HVEM-BTLA signaling axis resulting in excessive production of pro-inflammatory cytokines, particularly those that promote neutrophil recruitment and activation such as IL-17 (78). Additionally, the absence of BTLA-mediated inhibition could also prolong the survival of activated CD4^+^ T cells, potentially maintaining a chronic inflammatory state in the airways (79). Therefore, we reasoned that inducing BTLA inhibitory signaling with a BTLA agonist may alleviate steroid-resistant airway inflammation and AHR as previously demonstrated in experimental asthma models (73). We found that BXD75 mice treated with BTLA agonist showed improvements in AHR, reduced pulmonary neutrophil number and frequencies, and decreased levels of IL-17A, IL-6 and CXCL1. The BTLA agonist treatment also attenuated the levels of Th17 cytokines and the Th2 cytokine, IL-5, in both BXD75 and C57BL/6 mice, consistent with previous findings in BTLA-deficient mice (22). While the exact mechanism of action across all cell types expressing BTLA remains to be fully elucidated, these results suggest that BTLA agonism could be a promising approach for managing neutrophilic asthma and other inflammatory conditions. In a mechanistic approach, we found that BTLA agonist treatment significantly reduced the number of Th17 cells and levels of IL-17 *ex vivo*, likely through the inhibition of both the canonical and non-canonical NF-κB pathways which are crucial for Th17 differentiation (72). This suggests that not only can BTLA control Th2 and Th17 inflammatory responses in asthma, but it can also modulate Th17 differentiation.

Our study proposes specifically targeting CD4^+^ T cells as a therapeutic strategy to attenuate AHR and lung inflammation in steroid-resistant asthma. However, targeting CD4^+^ T cells in mice and patients presents significant challenges and warrants further investigation. The intricate interplay between LIGHT-HVEM-BTLA signaling on CD4^+^ T cells may play a crucial role in the development of severe steroid-resistant asthma in BXD75 mice. High levels of HVEM and LIGHT and concurrent low levels of BTLA on CD4^+^ T cells may exacerbate the inflammatory response in several ways. Without sufficient BTLA to engage HVEM, this receptor may become more available for interactions with other binding partners, such as LIGHT, which could further enhance T cell activation. Another limitation of our study is that we did not investigate the blocking of HVEM. Since HVEM functions as a bidirectional molecular switch, mediating both inhibitory and stimulatory pathways, blocking HVEM signaling could potentially disrupt this balance. Therefore, future studies should focus on selectively blocking HVEM pro-inflammatory signaling and assess the impact on the establishment and exacerbation of steroid-resistant airway inflammation. Nevertheless, our findings illuminate the promising potential of leveraging inhibitory signaling to counteract excessive inflammation in steroid-resistant asthma.

Lastly, it is important to note that our study exclusively involves female mice. Asthma is known to exhibit sex-based differences in presentation due to factors such as hormones, airway anatomy and lifestyle choices influenced by gender (80, 81). Adult females are more likely to develop severe asthma (81).We focused exclusively on female mice in this study to model severe asthma as they might better represent certain aspects of severe asthma observed in females (80, 81). While our findings provide valuable insights into the mechanisms of airway inflammation and BTLA signaling, future research incorporating both male and female mice will be crucial to allow for a more comprehensive understanding of these processes and ensure that our findings are broadly applicable. Lastly, the type of allergen and adjuvant used may also impact inflammatory responses.

In conclusion, we have identified in this study that the BXD75 mouse strain recapitulates the characteristics of steroid-resistant asthma in humans when challenged with HDM. This strain has provided valuable insights into the therapeutic potential of inducing BTLA inhibitory signaling as a treatment strategy for steroid-resistant asthma. Previous studies have highlighted a role for BTLA in airway inflammation, but to the best of our knowledge, our study demonstrates for the first time the role of BTLA in steroid-resistant asthma through a mechanism by which BTLA enhancement attenuates Th17 number and activity by downregulating NF-κB signaling.

## Materials and methods

### Mice

C57BL/6 (Strain #000664) and 58 mice strains (**Supplementary Table 1**) were purchased from Jackson Laboratory (Bar Harbor, ME). Six to eight-week-old aged- female mice were used in the study. Mice were maintained and bred in the pathogen-free animal facility at the Keck School of Medicine, University of Southern California (USC). All animal studies were approved by the USC institutional Animal Care and Use Committee (IACUC) and conducted in accordance with the USC Department of Animal Resources’ guidelines.

### *In vivo* murine experiments

House dust mite extract from *D. Pteronyssinus* (Cat# XPB82D3A25, Lot#145793, Greer Laboratories, Lenoir, NC) were purchased in large bulk, aliquoted and stored at -80°C. Mice were immunized with 25 μg of house dust mite in 0.5 mg alum (Alhydrogel^®^ adjuvant 2%, InvivoGen). 7 days later, mice were anesthetized with combination of 80-100 mg/kg mouse of ketamine + 5-10 mg/kg xylazine *i.p.* and then were challenged with 50 μg of house dust mite in 40 μl of PBS or PBS only intranasally (*i.n*.) over 3 consecutive days. For studying steroid resistance, mice were also injected intraperitoneally (*i.p*.) with 1 mg/kg mouse dexamethasone or vehicle on the same 3 consecutive days of intranasal house dust mite challenge as previously described (34). For studying CD4 and CD8 T cell depletion, mice were injected i.p. with 500 μg of anti-CD4 (Clone GK1.5, BioXCell) or anti-CD8 (Clone 53-6.7, BioXCell) or isotype 24 hours before the first intranasal HDM challenge. 24 hours after the last intranasal challenge, mice were anesthetized with combination of 80-100 mg/kg mouse of ketamine + 5-10 mg/kg xylazine *i.p.*, and lung function and BALF cellularity was measured. After AHR and BALF were acquired, mice were euthanized by cervical dislocation. For studying BTLA agonism, mice were injected *i.p*. with 100 μg BTLA agonist (Clone 6A6, BioXCell) or isotype on the same 3 consecutive days of intranasal HDM challenge. 24 hours after the last intranasal challenge, lung function was measured, BALF and lungs were collected for downstream analysis.

### Measurement of airway hyperreactivity

Lung function was assessed by direct measurement of lung resistance and dynamic compliance (cDyn) in restrained, tracheostomized, and mechanically ventilated mice using the FinePointe RC system (Buxco Research Systems) using FinePointe software v2.4.4.9183 under general anesthesia as previously described (28, 82). Mice were sequentially challenged with aerosolized PBS (baseline), followed by increasing doses of methacholine ranging from 5 to 80 mg/mL (82, 83). The methacholine is nebulized over a period of 30 seconds and delivered to the mouse over a period of 120 seconds followed by a 30 second period to return to baseline per each methacholine dose. The entire process per mouse takes approximately 20 minutes including an initial acclimation period and a baseline PBS challenge period. Lung resistance and dynamic compliance are recorded every 2 seconds. AHR data were analyzed by repeated measurements of a general linear model.

### Tissue processing and flow cytometry

Following AHR measurements, the trachea was cannulated and the BALF was collected. Briefly, lungs were washed three times with 1 mL of ice-cold PBS and collected into a single tube. The BALF was centrifuged at 500 x g for 6 min at 4°C. The supernatant was collected into a separate tube for downstream cytokine analysis. The resultant pellet was resuspended in 1X RBC lysis buffer (Biolegend) and incubated at room temperature for 5 min to lyse red blood cells (RBCs). The reaction was terminated by washing the cells with PBS. The remaining pellet was then stained for flow cytometry. Immune cell populations were identified in BALF using the following antibodies: APC-Cy7 anti-mouse CD45 (clone 30-F11; BioLegend), PE-Cy7 anti-mouse CD11c (clone HL3; BD Bioscences), PE anti-mouse Siglec-F (clone E50-2440; BD Biosciences), BV421 anti-mouse/human CD11b (clone M1/70, BioLegend), APC anti-mouse Ly6G (clone 1A8, BioLegend), BV510 anti-mouse CD19 (clone 6D5; BioLegend), PerCP/Cyanine5.5 anti-mouse CD3 (clone 17A2; BioLegend), and anti-mouse Fc-block (2.4G2. BioXcell). CountBright Absolute Count Beads (Thermo Fisher Scientific, Waltham, MA) were used to count BALF and lung immune cells.

Lungs were collected and minced into small pieces and subsequently incubated in type IV collagenase (400 U/ml; Worthington Biochemicals) at 37 °C for 60 min. After digestion, the lung digest was passed through a 70-μm cell strainer (Falcon) to create a single cell suspension. Cells were washed with PBS and centrifuged at 500 x g for 6 min at 4°CC. The resultant pellet was resuspended in 1X RBC lysis buffer (Biolegend) and incubated at room temperature for 5 min to lyse red blood cells (RBCs). The cells were washed with PBS. The remaining pellet was then further prepared for flow cytometry or FACS sorting. Cells in the lungs were stained for LIGHT/HVEM/BTLA with the following antibodies: FITC anti-mouse CD45 (clone 30-F11, eBioscience), APC-Cy7 anti-mouse CD11c (clone N418, BioLegend), PerCP/Cyanine5.5 anti-mouse CD3 (clone 17A2; BioLegend), BV510 anti-mouse CD4 (clone RM4-5, BioLegend), PE-Cy7 anti-mouse CD8a (clone 53-6.7, BioLegend), Alexa Fluor^®^ 647 anti-mouse LIGHT (clone 885310, R&D Systems), APC anti-mouse HVEM (clone HMHV-1B18, BioLegend), APC anti-mouse BTLA (clone 6A6, BioLegend). For *ex vivo* experiments, intracellular staining was performed using the BD Cytofix/Cytoperm kit (BD Biosciences) and used according to the manufacturer’s instructions. Cells were stimulated 4 hrs with 50 mg/mL PMA (Sigma-Aldrich), 500 mg/mL ionomycin (Sigma-Aldrich), and 1 mg/mL GolgiStop (BD Biosciences). Cells were then stained with PerCP/Cyanine5.5 anti-mouse CD3 (clone 17A2; BioLegend), BV510 anti-mouse CD4 (clone RM4-5, BioLegend), PE anti-mouse IL-17A (Clone eBio17B7, eBioscience). Staining for the NFkB pathway was done with the following antibodies: PE-anti-mouse TRAF2 (Clone H-10, Santa Cruz Biotechnology), PE anti-mouse NIK (Clone A-12, Santa Cruz Biotechnology), Alexa^®^ Fluor 647 anti-mouse NFκB p52/p100/NFKB2 Antibody (Clone C-5, Santa Cruz Biotechnology), PE anti-human/mouse p65/RelA (Clone 532301, R&D Systems). Staining for BTLA signaling was done with the following antibodies: PE anti-human/mouse SHP-1 (Clone Y476, Abcam), APC anti-human/mouse pAkt (S473) (Clone SDRNR, eBioscience).Cells were stained for FACS sorting with APC-Cy7 anti-mouse CD45 (clone 30-F11; BioLegend), PerCP/Cyanine5.5 anti-mouse CD3 (clone 17A2; BioLegend), and BV510 anti-mouse CD4 (clone RM4-5, BioLegend).

A 1:500 dilution was used for all antibodies except for a 1:150 dilution for intracellular staining. Acquisition was performed on a BD FACSCanto II (BD Biosciences) using the BD FACSDiva software v8.0.1. Data were analyzed with FlowJo software (TreeStar) version 10.

### Cytokine measurements

BALF supernatant and cell culture media were collected to quantify cytokines using the Mouse Th Cytokine Panel (12-plex) w/VbP V03 LEGENDplex™ Multiplex Assay (Biolegend) according to manufacturer’s instructions (84). The following cytokines were measured: IL-2, IL-4, IL-5, IL-6, IL-9, IL-10, IL-13, IL-17A, IL-17F, IL-22, TNF- α, and IFN-γ. CXCL1 (KC) levels were measured by enzyme-linked immunosorbent assay kit (ELISA) purchased from BioLegend. Data were collected acquired with Attune™ Cytometric Software v5.3.0 from the Attune NxT (Life Technologies). Data were analyzed with LEGENDplex Data Analysis Software Suite (BioLegend).

### Lung histology

After euthanasia mice lungs were harvested and fixed in 10% neutral buffered formalin solution. The same lung lobe was consistently used for analysis across all mice in the study. The lungs were embedded in paraffin and cut into 3 serial sections at a thickness of 5 μm. Tissue sections were routinely stained with hematoxylin and eosin (H&E) and Periodic acid-Schiff (PAS) stain. Neutrophils were identified by their multilobed nucleus and light staining cytoplasm. Eosinophils were identified by their dark pink staining cytoplasmic granules. Images were acquired on a Keyence BZ-X710 (Keyence, Itasca, IL) using a 40x and 20x objective using BZ-X-Viewer and analyzed with BZ-X-Analyzer v1.3.0.3 software.

### RNA-sequencing

CD45^+^CD3^+^CD4^+^ T cells were sorted on the FACSARIA III system (BD Biosciences) from the lungs of mice challenged on 3 consecutive days i.n. with HDM. Total RNA was isolated using the RNeasy Mini Kit (QIAGEN) according to the manufacturer’s instructions. For each sample, 10 ng of input RNA was used to produce cDNA for downstream library preparation. Samples were sequenced on a NextSeq 500 (Illumina) system. Raw reads were aligned, normalized, and further analyzed using Partek Flow, version 12.2 Copyright; Partek Inc. Normalized read counts were tested for differential expression using Partek’s Gene-Specific Analysis (GSA) algorithm. Comparative functional enrichment analysis was performed with Ingenuity Pathway Analysis (IPA) (QIAGEN) The data discussed in this publication has been deposited in NCBI’s Gene Expression Omnibus and are accessible through GEO series accession number GSE284206.

### *In vitro* Th17 differentiation

Naïve CD4 T cells from C57BL/6 and BXD75 mice were isolated by magnetic bead enrichment using the mouse CD4 (L3T4) microbeads (Miltenyl Biotec) according to the manufacturer’s protocol on the AutoMACS Pro Separator (Miltenyl Biotec). Naïve CD4 T cells were cultured in complete RPMI media (RPMI 1640 with 10% heat inactivated FBS, 100 U/mL penicillin and 100 mg/ml streptomycin) with plate-bound 10 μg/mL anti-CD3 (Clone 17A2, BioXCell). For Th17 differentiation, naive CD4^+^ T cells were stimulated with 5 μg/mL soluble anti-CD28 (Clone 37.51, eBioscience), 10 ng/mL rmIL-6 (R&D Systems), 5 ng/mL rmTGF-b1 (BioLegend), 10 ng/mL rmIL-23 (BioLegend), 10 μg/mL mouse anti-IL-12 p75 (Clone R2-9A5), 10 μg/mL mouse anti-IFN-γ (Clone XMG1.2, BioLegend) and 10 μg/mL mouse anti-IL-4 (Clone 11B11, BioLegend). For BTLA agonist treatment, 20 μg/mL of BTLA agonist was added in the culture on Day 3 of stimulation. On Day 5, T cells were collected and analyzed.

### Quantification and statistical analysis

Data are representative of at least two independent experiments or pooled from two experiments. Data are presented as means ± SEM (except for RNAseq). For multigroup comparisons, we used two-way ANOVA with Tukey’s multiple comparison test. Data were analyzed with Prism 10 Software v10.4.0 (GraphPad Software Inc.). p value < 0.05 was considered to denote statistical significance (*p <0.05, **p < 0.01, ***p < 0.001, ****p<0.0001).

## Data Availability

The datasets presented in this study can be found in online repositories. The names of the repository/repositories and accession number(s) can be found below: GSE284206 (GEO).
