# Supplementary material for: BTLA agonist attenuates Th17-driven inflammation in a mouse model of steroid-resistant asthma
Source: Front Immunol. 2025 Mar 28;16:1552394. doi: 10.3389/fimmu.2025.1552394 (PMC11986467; doi:10.3389/fimmu.2025.1552394)
Supplement: Supplementary file 1 [file DataSheet1.docx]

Supplementary Material

Fig. S1


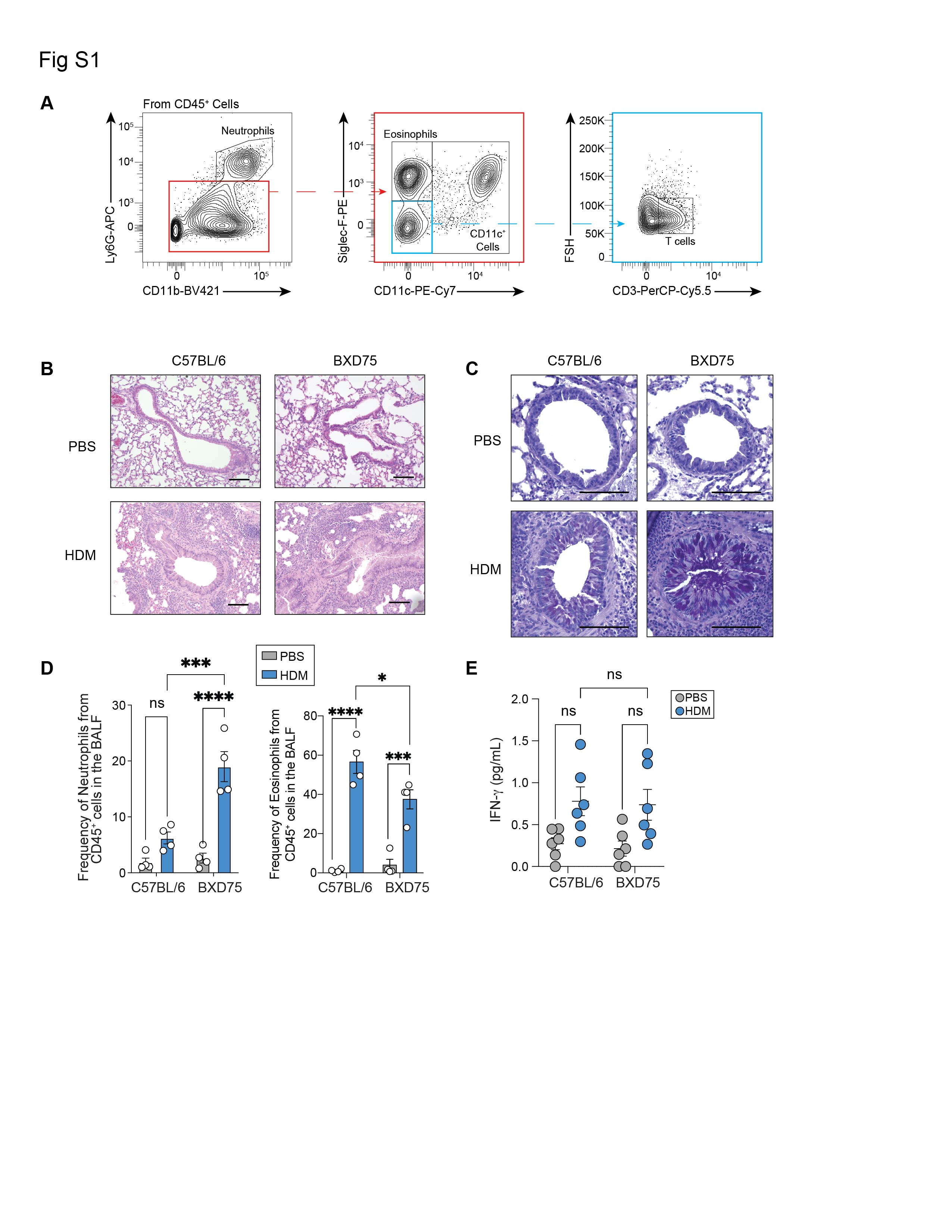


**Fig. S1** **House dust mite induces an increase in pro-inflammatory immune cells in the BALF.**

**(A**) Representative gating strategy for CD45^+^ cells, CD11c^+^ cells, neutrophils (Ly6G^+^CD11b^+^), eosinophils (Siglec-F^+^, CD11c^-^), and T cells (CD3^+^) in BALF of mice exposed to HDM. (**B**) H&E staining of lung sections at 20x magnification. Scale bar=100 μm. (**C**) Periodic acid-Schiff (PAS) staining of lung sections at 40x magnification. Scale bar=100 μm. (**D**) Frequency of neutrophils and eosinophils from CD45^+^ cells in the BALF. (**E**) Levels of IFNγ in the BALF. For all quantifications, data are presented as mean + SEM and analyzed with a two-way ANOVA with Tukey’s multiple comparison test. n.s., not significant; *, *p* < 0.05; **, *p* < 0.01; ***, *p* < 0.001; ***, *p* < 0.0001.

Fig. S2


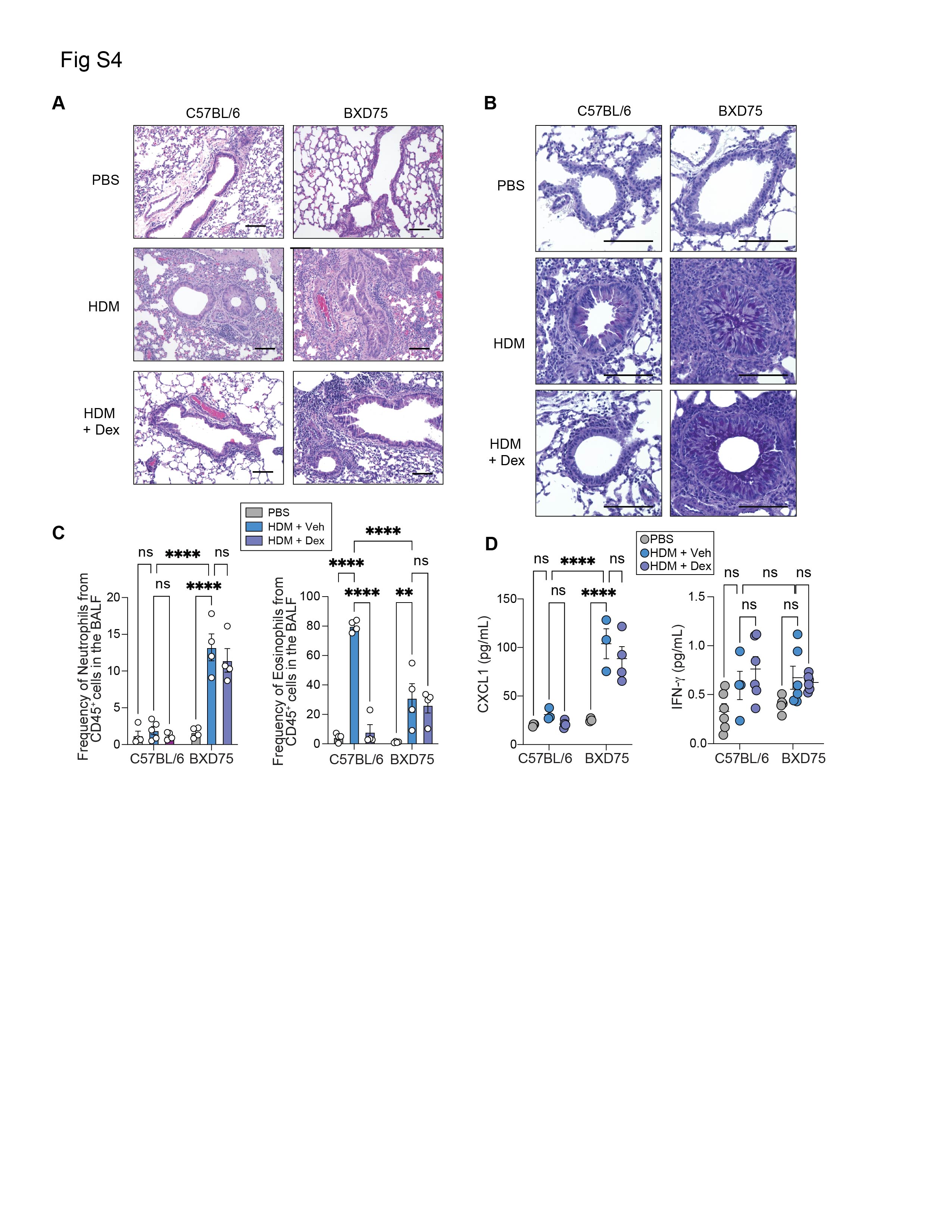


**Fig. S2 Dexamethasone treatment does not affect goblet cell hyperplasia and inflammation in BXD75 mice.**

(**A**) H&E staining of lung sections at 20x magnification. Scale bar=100 μm. (**B**) Periodic acid-Schiff (PAS) staining of lung sections at 40x magnification. Scale bar=100 μm. (**C**) Frequency of neutrophils and eosinophils from CD45^+^ cells in the BALF. (**D**) Levels of IFNγ in the BALF. Dex, dexamethasone. Veh, vehicle. For all quantifications, data are presented as mean + SEM and analyzed with a two-way ANOVA with Tukey’s multiple comparison test. n.s., not significant; *, *p* < 0.05; **, *p* < 0.01; ***, *p* < 0.001; ***, *p* < 0.0001.

Fig. S3


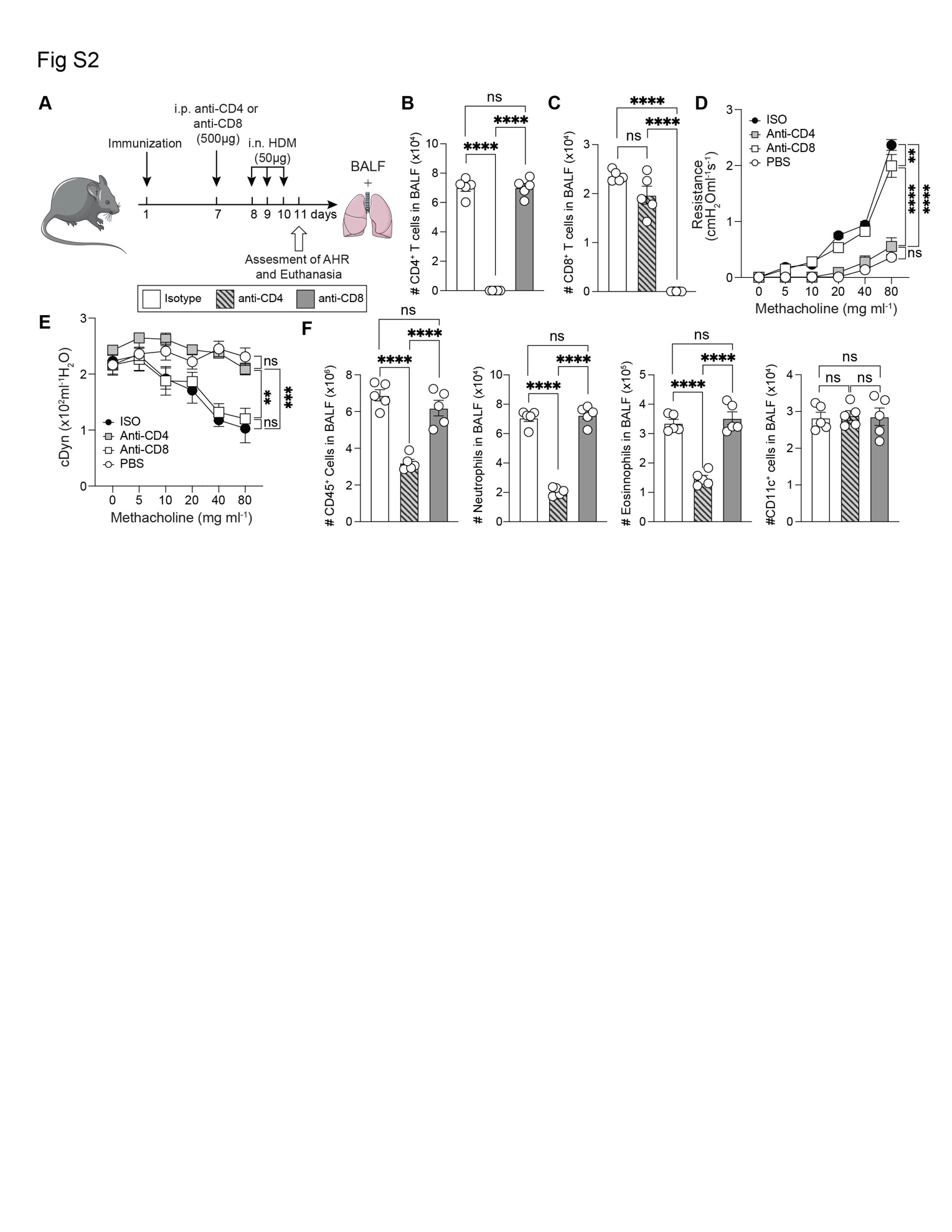


**Fig. S3** **Depletion of CD4^+^ T cells during HDM challenge ameliorates AHR and lung inflammation.**

**(A**) C57BL/6 mice were immunized with 25 μg of HDM in 0.5 mg Alum. On Day 7, mice were administered intraperitoneally (*i.p*.) with 500 μg anti-CD4, anti-CD8 or isotype antibodies. On Day 8, mice were intranasally (*i.n*.) challenged with 50 μg of HDM or PBS for 3 consecutive days. On Day 11, lung function (AHR) and BALF cellularity were assessed. Total number of (**B**) CD4^+^ T cells (CD45^+^, CD3^+^, CD4^+^) and (**C**) CD8^+^ T cells (CD45^+^, CD3^+^, CD8^+^) in the BALF after anti-CD4, anti-CD8, or isotype treatment. (**D**) Lung resistance and (**E**) dynamic compliance of C57BL/6 mice challenged with HDM over 3 consecutive days. (**F**) Total number of CD45^+^ cells, neutrophils (CD45^+^, Ly6G^+^, CD11b^+^), eosinophils (CD45^+^, CD11c^-^, Siglec-F^+^), and CD11c^+^ cells (CD45^+^, Ly6G^-^, CD11c^+^) in the BALF. Data are representative of at least two experiments. For all experiments n = 5 mice per group. For all quantifications, data are presented as mean + SEM and analyzed with a one-way ANOVA with Tukey’s multiple comparison test. n.s., not significant; *, *p* < 0.05; **, *p* < 0.01; ***, *p* < 0.001; ***, *p* < 0.0001.

Fig. S4

**
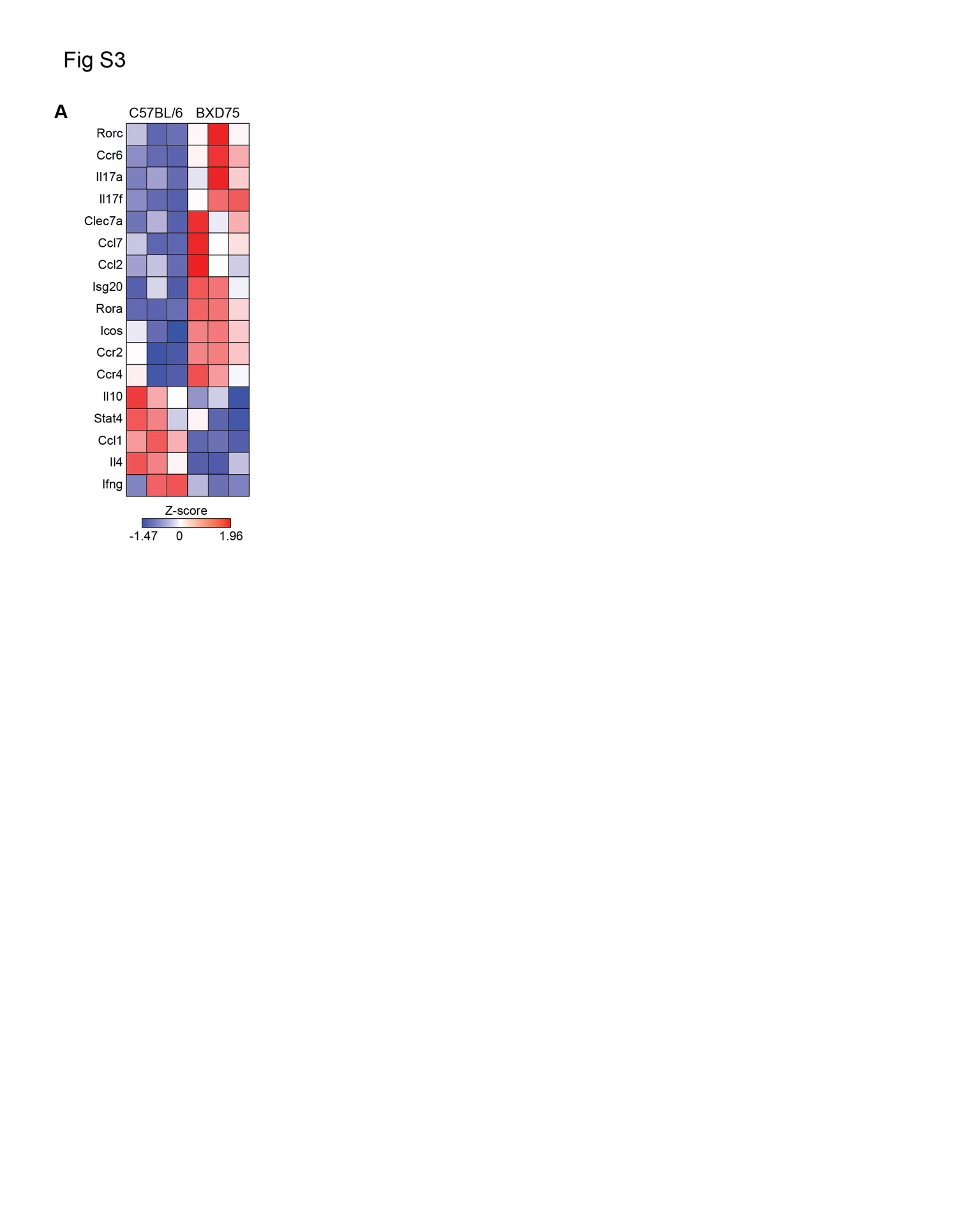
**

**Fig. S4 Transcription factors driving Th17 differentiation are differentially expressed in CD4^+^ T cells from HDM-challenged BXD75 mice.**

Heatmap representation of transcription factors driving Th17 differentiation in CD4^+^ T cells isolated from BXD75 mice and C57BL/6 mice exposed to HDM.

Fig. S5


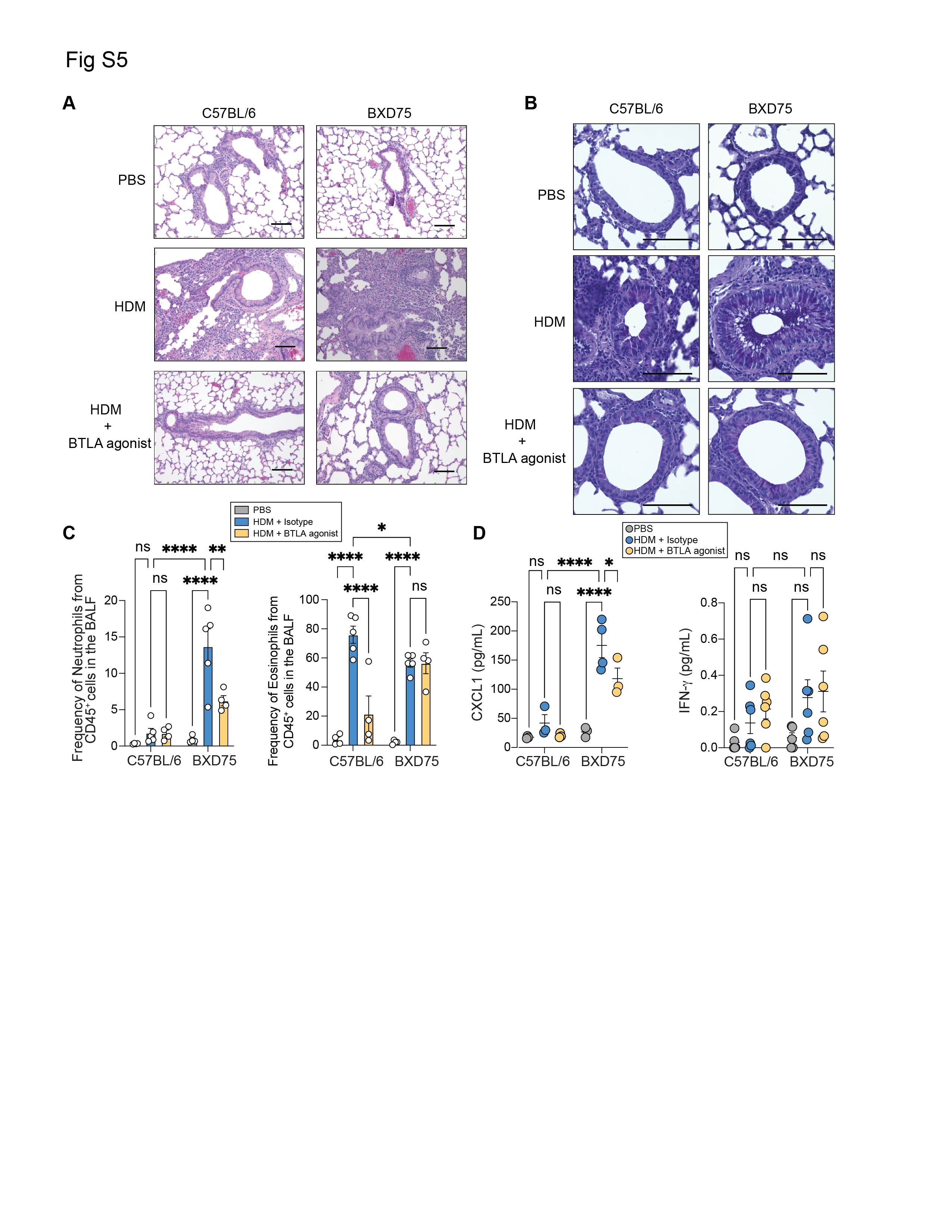


**Fig. S5 BTLA agonist improves goblet cell hyperplasia in HDM challenged mice.**

(**A**) H&E staining of lung sections at 20x magnification. Scale bar=100 μm. (**B**) Periodic acid-Schiff (PAS) staining of lung sections at 40x magnification. Scale bar=100 μm. (**C**) Frequency of neutrophils and eosinophils from CD45^+^ cells in the BALF. (**D**) Levels of IFNγ in the BALF. For all quantifications, data are presented as mean + SEM and analyzed with a two-way ANOVA with Tukey’s multiple comparison test. n.s., not significant; *, *p* < 0.05; **, *p* < 0.01; ***, *p* < 0.001; ***, *p* < 0.0001.

Fig. S6


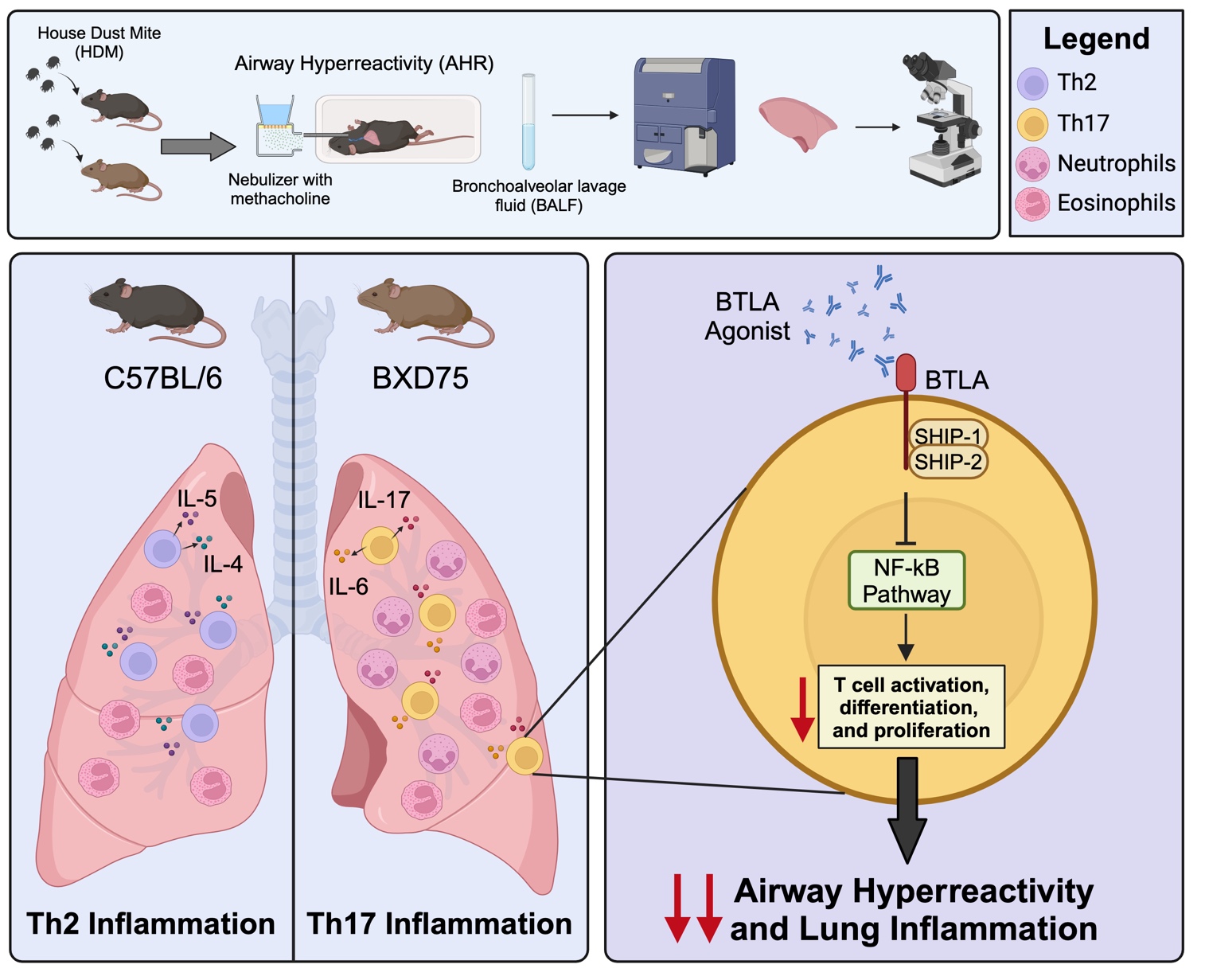


**Fig. S6 BTLA agonist attenuates Th17 inflammation in a mouse model of steroid-resistant asthma**

Graphical abstract illustrating the key methodologies in investigating HDM-induced asthma including assessment of AHR, flow cytometry analysis of BALF and lung histology. The inflammatory response of C57BL/6 and BXD75 mice are compared following HDM exposure and the mechanism by which BTLA agonist therapy alleviates AHR and lung inflammation are also depicted.

**Table S1.** **List of 58 mouse strains used in the study.**

| Strain | Stock # | Strain | Stock # | Strain | Stock # |
| --- | --- | --- | --- | --- | --- |
| AKR/J | 000648 | BXD40/TyJ | 003229 | BXH6/TyJ | 000038 |
| AXB12/PgnJ | 001683 | BXD42/TyJ | 003230 | C3H/HeJ | 000659 |
| AXB13/PgnJ | 001826 | BXD43/RwwJ | 007093 | C57BL/6J | 000664 |
| AXB19/PgnJ | 001687 | BXD44/RwwJ | 007094 | C57BLKS/J | 000662 |
| AXB23/PgnJ | 001690 | BXD45/RwwJ | 007096 | C57L/J | 000668 |
| AXB24/PgnJ | 001691 | BXD49/RwwJ | 007098 | C58/J | 000669 |
| AXB4/PgnJ | 001676 | BXD5/TyJ | 000037 | CBA/J | 000656 |
| AXB5/PgnJ | 001677 | BXD50/RwwJ | 007099 | DBA/2J | 000671 |
| AXB6/PgnJ | 001678 | BXD56/RwwJ | 007104 | I/LnJ | 000674 |
| AXB8/PgnJ | 001679 | BXD61/RwwJ | 007106 | LP/J | 000676 |
| BALB/cByJ | 001800 | BXD64/RwwJ | 007109 | NZB/BinJ | 000684 |
| BALB/cJ | 000651 | BXD66/RwwJ | 007111 | NZW/LacJ | 001058 |
| BXA1/PgnJ | 001692 | BXD67/RwwJ | 007112 | PL/J | 000680 |
| BXA11/PgnJ | 001699 | BXD68/RwwJ | 007113 | RIIIS/J | 000683 |
| BXA12/PgnJ | 001700 | BXD70/RwwJ | 007115 | SEA/GnJ | 000644 |
| BXA2/PgnJ | 001693 | BXD75/RwwJ | 007119 | SJL/J | 000686 |
| BXA25/PgnJ | 001711 | BXD77/RwwJ | 007121 | SM/J | 000687 |
| BXD13/TyJ | 000040 | BXD87/RwwJ | 007130 | SWR/J | 000689 |
| BXD20/TyJ | 000330 | BXD9/TyJ | 000105 |  |  |
| BXD39/TyJ | 003228 | BXD98/RwwJ | 007141 |  |  |

**Table S2. Raw data values for radial graphs presented in Figure 1.**

| Strain | Treatment | Neutrophil Count | Lung Resistance (cmH_2_Oml^-1^s^-1^) | IgE (pg/mL) |
| --- | --- | --- | --- | --- |
| AKR/J | PBS | 106.796 | 2.43554 | 1661.32 |
|  | HDM | 1181.78 | 5.0689 | 4307.98 |
|  | Δ (HDM-PBS) | 1074.98028 | 2.63336561 | 2646.65887 |
| AXB12/PgnJ | PBS | 19.7865 | 1.86935 | 4862.62 |
|  | HDM | 5627.22 | 4.89095 | 5556.93 |
|  | Δ (HDM-PBS) | 5607.43303 | 3.02159504 | 694.313199 |
| AXB13/PgnJ | PBS | 450.869 | 1.84822 | 3466.83 |
|  | HDM | 4772.26 | 3.25796 | 10543.6 |
|  | Δ (HDM-PBS) | 4321.39533 | 1.40974557 | 7076.79617 |
| AXB19/PgnJ | PBS | 122.436 | 1.6378 | 4554.06 |
|  | HDM | 595.948 | 3.54484 | 26070.7 |
|  | Δ (HDM-PBS) | 473.512093 | 1.90703285 | 21516.6288 |
| AXB23/PgnJ | PBS | 197.747 | 1.99594 | 153.754 |
|  | HDM | 49070.8 | 3.60377 | 562.874 |
|  | Δ (HDM-PBS) | 48873.0932 | 1.60783132 | 409.120093 |
| AXB24/PgnJ | PBS | 7.54894 | 1.59142 | 686.88 |
|  | HDM | 2284.22 | 3.81004 | 692.776 |
|  | Δ (HDM-PBS) | 2276.67381 | 2.21862491 | 5.89609157 |
| AXB4/PgnJ | PBS | 11.0901 | 2.3949 | 142.677 |
|  | HDM | 853.128 | 4.66974 | 351.534 |
|  | Δ (HDM-PBS) | 842.03828 | 2.27484728 | 208.85695 |
| AXB5/PgnJ | PBS | 275.409 | 2.38284 | 878.444 |
|  | HDM | 2395.82 | 6.69151 | 907.094 |
|  | Δ (HDM-PBS) | 2120.41133 | 4.3086741 | 28.6502489 |
| AXB6/PgnJ | PBS | 210.671 | 2.15705 | 838.701 |
|  | HDM | 38119.8 | 5.65684 | 1428.69 |
|  | Δ (HDM-PBS) | 37909.1287 | 3.49979538 | 589.990966 |
| AXB8/PgnJ | PBS | 50.1933 | 1.50336 | 1128.86 |
|  | HDM | 12044.6 | 3.34725 | 1931.73 |
|  | Δ (HDM-PBS) | 11994.3743 | 1.84388932 | 802.874191 |
| BALB/cByJ | PBS | 80.2396 | 2.0736 | 3014.31 |
|  | HDM | 1032.14 | 8.24897 | 10667.2 |
|  | Δ (HDM-PBS) | 951.896353 | 6.17537636 | 7652.91424 |
| BALB/cJ | PBS | 46.2539 | 2.98075 | 1593.68 |
|  | HDM | 810.129 | 5.9677 | 10667.2 |
|  | Δ (HDM-PBS) | 763.875493 | 2.9869489 | 9073.54751 |
| BXA1/PgnJ | PBS | 143.651 | 1.16488 | 579.8 |
|  | HDM | 9504.05 | 2.53406 | 772.917 |
|  | Δ (HDM-PBS) | 9360.39948 | 1.36917935 | 193.117719 |
| BXA11/PgnJ | PBS | 39.7454 | 1.23065 | 3770.68 |
|  | HDM | 840.696 | 4.23436 | 6595.7 |
|  | Δ (HDM-PBS) | 800.950703 | 3.00371033 | 2825.02484 |
| BXA12/PgnJ | PBS | 17.0834 | 0.49764 | 948.338 |
|  | HDM | 1525.19 | 4.02354 | 1127.48 |
|  | Δ (HDM-PBS) | 1508.10281 | 3.525903 | 179.145757 |
| BXA2/PgnJ | PBS | 107.751 | 1.80331 | 242.633 |
|  | HDM | 59399.9 | 3.61259 | 362.889 |
|  | Δ (HDM-PBS) | 59292.1956 | 1.80928408 | 120.256433 |
| BXA25/PgnJ | PBS | 1.63287 | 2.19568 | 3177.9 |
|  | HDM | 32.4063 | 4.38636 | 7685.18 |
|  | Δ (HDM-PBS) | 30.7734583 | 2.1906779 | 4507.28156 |
| BXD13/TyJ | PBS | 109.838 | 1.9505 | 1522.28 |
|  | HDM | 164.813 | 4.21061 | 1595.02 |
|  | Δ (HDM-PBS) | 54.9744452 | 2.26010672 | 72.7421287 |
| BXD20/TyJ | PBS | 192.942 | 2.39051 | 1040.96 |
|  | HDM | 5450.67 | 3.48134 | 2193.19 |
|  | Δ (HDM-PBS) | 5257.72969 | 1.09082268 | 1152.22153 |
| BXD39/TyJ | PBS | 104.514 | 2.14913 | 569.232 |
|  | HDM | 507.878 | 4.61089 | 1071.44 |
|  | Δ (HDM-PBS) | 403.364086 | 2.46175665 | 502.212304 |
| BXD40/TyJ | PBS | 65.4969 | 1.82219 | 222.453 |
|  | HDM | 1504.58 | 2.93451 | 277.03 |
|  | Δ (HDM-PBS) | 1439.08157 | 1.11232317 | 54.5762318 |
| BXD42/TyJ | PBS | 41.3482 | 3.50117 | 231.221 |
|  | HDM | 9608.25 | 3.80058 | 411.85 |
|  | Δ (HDM-PBS) | 9566.90608 | 0.29940541 | 180.628228 |
| BXD43/RwwJ | PBS | 25.1223 | 1.833 | 3761.95 |
|  | HDM | 356.468 | 3.53129 | 4368.01 |
|  | Δ (HDM-PBS) | 331.345984 | 1.69828741 | 606.059532 |
| BXD44/RwwJ | PBS | 36.1317 | 2.09939 | 1328.38 |
|  | HDM | 168.577 | 3.93781 | 2195.62 |
|  | Δ (HDM-PBS) | 132.444964 | 1.83842338 | 867.231397 |
| BXD45/RwwJ | PBS | 114.272 | 1.65759 | 317.038 |
|  | HDM | 1485.44 | 3.59827 | 484.408 |
|  | Δ (HDM-PBS) | 1371.17253 | 1.94067942 | 167.370039 |
| BXD49/RwwJ | PBS | 5.18067 | 1.34609 | 378.061 |
|  | HDM | 155.378 | 2.80704 | 384.367 |
|  | Δ (HDM-PBS) | 150.196884 | 1.46095717 | 6.30609769 |
| BXD5/TyJ | PBS | 20.7572 | 1.68819 | 1113.93 |
|  | HDM | 2344.29 | 4.92008 | 1141.64 |
|  | Δ (HDM-PBS) | 2323.53221 | 3.23189216 | 27.7145007 |
| BXD50/RwwJ | PBS | 35.0328 | 1.53754 | 949.426 |
|  | HDM | 505.641 | 2.74556 | 1275.5 |
|  | Δ (HDM-PBS) | 470.607761 | 1.20801429 | 326.078239 |
| BXD56/RwwJ | PBS | 20.3531 | 1.59287 | 151.996 |
|  | HDM | 338.553 | 2.61681 | 184.226 |
|  | Δ (HDM-PBS) | 318.199883 | 1.02394169 | 32.2291975 |
| BXD61/RwwJ | PBS | 3.89004 | 1.73558 | 1223.84 |
|  | HDM | 47516.7 | 4.84943 | 1397.42 |
|  | Δ (HDM-PBS) | 47512.8346 | 3.11385238 | 173.577751 |
| BXD64/RwwJ | PBS | 1032.23 | 1.83263 | 5138.76 |
|  | HDM | 40029.5 | 3.68806 | 9403.38 |
|  | Δ (HDM-PBS) | 38997.2449 | 1.85543796 | 4264.61966 |
| BXD66/RwwJ | PBS | 589.632 | 1.51853 | 112.071 |
|  | HDM | 2160.17 | 2.55834 | 271.39 |
|  | Δ (HDM-PBS) | 1570.53293 | 1.03981139 | 159.318937 |
| BXD67/RwwJ | PBS | 4.94592 | 1.22577 | 1411.46 |
|  | HDM | 5079.74 | 4.14304 | 1535.01 |
|  | Δ (HDM-PBS) | 5074.79609 | 2.91726452 | 123.553724 |
| BXD68/RwwJ | PBS | 164.218 | 1.70217 | 1732.91 |
|  | HDM | 47339.7 | 3.595 | 3105.71 |
|  | Δ (HDM-PBS) | 47175.4954 | 1.89283288 | 1372.80471 |
| BXD70/RwwJ | PBS | 91.7234 | 1.48093 | 363.581 |
|  | HDM | 493.698 | 3.49962 | 579.91 |
|  | Δ (HDM-PBS) | 401.974744 | 2.01869094 | 216.329645 |
| BXD75/RwwJ | PBS | 1397.02 | 1.82977 | 344.628 |
|  | HDM | 84389.6 | 4.92764 | 965.403 |
|  | Δ (HDM-PBS) | 82992.5662 | 3.09787137 | 620.775356 |
| BXD77/RwwJ | PBS | 294.386 | 1.4086 | 827.184 |
|  | HDM | 25799.8 | 3.74618 | 1151.18 |
|  | Δ (HDM-PBS) | 25505.3776 | 2.33758048 | 323.991072 |
| BXD87/RwwJ | PBS | 691.268 | 1.88979 | 2648.21 |
|  | HDM | 1572.78 | 4.51935 | 2852.12 |
|  | Δ (HDM-PBS) | 881.508864 | 2.62955403 | 203.911552 |
| BXD9/TyJ | PBS | 431.378 | 1.78472 | 1705.48 |
|  | HDM | 25458.4 | 3.99439 | 3186.04 |
|  | Δ (HDM-PBS) | 25027.0122 | 2.20967198 | 1480.56073 |
| BXD98/RwwJ | PBS | 27.0929 | 2.3089 | 5351.43 |
|  | HDM | 244.668 | 4.79272 | 6244.71 |
|  | Δ (HDM-PBS) | 217.575479 | 2.48381914 | 893.284342 |
| BXH6/TyJ | PBS | 10.4095 | 2.18484 | 3087.61 |
|  | HDM | 127.878 | 4.91966 | 14059.6 |
|  | Δ (HDM-PBS) | 117.46858 | 2.7348132 | 10971.9568 |
| C3H/HeJ | PBS | 244.962 | 1.31292 | 3751.75 |
|  | HDM | 579.207 | 3.06328 | 4782.5 |
|  | Δ (HDM-PBS) | 334.245428 | 1.75036003 | 1030.75094 |
| C57BL/6J | PBS | 147.284 | 1.85319 | 1709.85 |
|  | HDM | 1687.74 | 4.30284 | 5510.48 |
|  | Δ (HDM-PBS) | 1540.45085 | 2.449654 | 3800.63134 |
| C57BLKS/J | PBS | 111.238 | 1.58572 | 1228.23 |
|  | HDM | 2146.77 | 4.28995 | 4859.43 |
|  | Δ (HDM-PBS) | 2035.53254 | 2.70422463 | 3631.19624 |
| C57L/J | PBS | 13.9032 | 3.33207 | 4189.01 |
|  | HDM | 939.653 | 5.31653 | 4321.97 |
|  | Δ (HDM-PBS) | 925.749947 | 1.98446149 | 132.96194 |
| C58/J | PBS | 117.117 | 1.49759 | 5491.68 |
|  | HDM | 12515.3 | 3.51279 | 12420.7 |
|  | Δ (HDM-PBS) | 12398.2253 | 2.01519833 | 6929.06393 |
| CBA/J | PBS | 524.556 | 1.95441 | 5495.63 |
|  | HDM | 848.953 | 4.46195 | 10255.7 |
|  | Δ (HDM-PBS) | 324.396506 | 2.50753332 | 4760.07352 |
| DBA/2J | PBS | 2138.15 | 1.62805 | 1016.21 |
|  | HDM | 3045.97 | 3.82201 | 1976.19 |
|  | Δ (HDM-PBS) | 907.821201 | 2.19395525 | 959.985929 |
| I/LnJ | PBS | 1321.61 | 1.97069 | 4170.93 |
|  | HDM | 1458.77 | 5.20851 | 4772.44 |
|  | Δ (HDM-PBS) | 137.154313 | 3.23781253 | 601.514913 |
| LP/J | PBS | 8.32655 | 2.81432 | 3278.68 |
|  | HDM | 73.8452 | 4.71573 | 15537.8 |
|  | Δ (HDM-PBS) | 65.5186557 | 1.90140863 | 12259.1023 |
| NZB/BinJ | PBS | 49.9566 | 1.58526 | 2925.47 |
|  | HDM | 7445.99 | 3.47719 | 3908.66 |
|  | Δ (HDM-PBS) | 7396.03564 | 1.89193469 | 983.184991 |
| NZW/LacJ | PBS | 306.163 | 1.50647 | 540.265 |
|  | HDM | 8933.95 | 2.70794 | 781.311 |
|  | Δ (HDM-PBS) | 8627.78673 | 1.20147466 | 241.046071 |
| PL/J | PBS | 380.212 | 1.67116 | 275.183 |
|  | HDM | 1161.59 | 4.22273 | 664.688 |
|  | Δ (HDM-PBS) | 781.382384 | 2.55157342 | 389.505503 |
| RIIIS/J | PBS | 2086.95 | 1.73721 | 555.86 |
|  | HDM | 8871.97 | 3.07307 | 683.139 |
|  | Δ (HDM-PBS) | 6785.01332 | 1.33585942 | 127.278584 |
| SEA/GnJ | PBS | 91.2238 | 2.24053 | 742.182 |
|  | HDM | 12605.1 | 4.3506 | 1418.42 |
|  | Δ (HDM-PBS) | 12513.8423 | 2.11007533 | 676.236032 |
| SJL/J | PBS | 2442.49 | 1.84912 | 433.295 |
|  | HDM | 43946.5 | 3.58464 | 993.784 |
|  | Δ (HDM-PBS) | 41504.0241 | 1.7355296 | 560.488466 |
| SM/J | PBS | 1395.9 | 2.4342 | 3319.29 |
|  | HDM | 4328.53 | 4.56884 | 4161.64 |
|  | Δ (HDM-PBS) | 2932.63498 | 2.13464729 | 842.357273 |
| SWR/J | PBS | 47.6381 | 0.95471 | 3086.06 |
|  | HDM | 1134.26 | 3.87252 | 19636.7 |
|  | Δ (HDM-PBS) | 1086.62324 | 2.91781305 | 16550.6746 |
